# Integrative Meta‐Analysis and WGCNA Reveal Candidate Diagnostic Hub Genes in Clear Cell Carcinoma

**DOI:** 10.1155/ijcb/5567255

**Published:** 2026-02-02

**Authors:** Haideh Namdari, Farhad Rezaei, Maryam Karamigolbaghi

**Affiliations:** ^1^ Iranian Tissue Bank and Research Center, Tehran University of Medical Sciences, Tehran, Iran, tums.ac.ir; ^2^ Virology Department, School of Public Health, Tehran University of Medical Sciences, Tehran, Iran, tums.ac.ir

**Keywords:** ccRCC, hub genes, meta-analysis, microarray, ROC analysis

## Abstract

Clear cell renal cell carcinoma (ccRCC) remains poorly understood at the genetic level. In this study, 12 GEO microarray datasets were combined, and meta‐analysis and multiparameter gene prioritization were used to reveal candidate diagnostic biomarkers. Improved preprocessing and analysis were used to identify differentially expressed genes (DEGs), which led to 4651 DEGs enriched in metabolic pathways, immune response, and kidney development. Key transcription factors (TFs), including ZNF692, ZNF395, and ZNF582, were identified. Utilizing WGCNA, 21 hub genes were identified, and diagnostic potential was determined via ROC analysis. Seventeen of the genes with AUC > 0.7 were validated as diagnostic hub genes, including ALDH2, ACADM, KIF11, and PTPRC. Nine of these genes showed significant prognostic relevance. The current work identifies key biomarkers that can enhance ccRCC diagnosis and guide future therapy.

## 1. Introduction

Renal cell carcinoma (RCC) causes 2%–3% of malignancies in adults and is the third leading cause of cancer‐related death among urologic tumors [1, 2]. Among RCC subtypes, clear cell renal cell carcinoma (ccRCC) constitutes 80%–90% of RCC [3, 4].

In spite of the progress in treatment modalities, the prognosis in advanced ccRCC is still poor, especially since metastatic ccRCC is resistant to chemotherapy as well as traditional radiotherapy. Traditional diagnostics with imaging and tissue biopsy are often not as sensitive and specific as required for early detection and proper prognosis [5].

Current studies have emphasized the potential of molecular biomarkers in optimizing the diagnosis and treatment of ccRCC. While genetic mutation and aberrant gene expression are both suspected to contribute to ccRCC pathogenesis, no conclusive diagnostic and prognostic biomarkers have yet been reported. Hence, a general understanding of the molecular pathophysiology of ccRCC and the determination of reliable biomarkers remain essential gaps in the area. This study is aimed at addressing these shortcomings through the conduct of a meta‐analysis of high‐throughput microarray data to identify differentially expressed genes (DEGs) in ccRCC. We employed weighted gene coexpression network analysis (WGCNA) in order to uncover gene modules and analyze their diagnostic potential. We cross‐validate these findings with the independent GSE40435 dataset, adding further strength to our candidate biomarkers.

## 2. Methods

### 2.1. Selection and Curation of Transcriptomic Datasets Pertaining to Renal Cell Carcinoma

We conducted an extensive search in the National Center for Biotechnology Information (NCBI) GEO database (http://www.ncbi.nlm.nih.gov/geo/) for studies that had described gene expression patterns in ccRCC. Search terms included “clear cell renal carcinoma,” refined by two filters: “Homo sapiens” and “Expression profiling by array.” As shown in Table 1, datasets were selected on the fact that they contained both tumor and normal tissue samples. We gathered data from the Affymetrix datasets GSE46699, GSE53757, GSE11151, GSE36895, GSE66270, GSE53000, GSE76351, GSE68417, and GSE11024 and Agilent datasets GSE16441, GSE71963, and GSE168845. In total, 271 normal tissue samples and 381 tumor tissue samples from 12 different ccRCC patient groups were used. The GSE40435 dataset, with 101 normal and 101 tumor samples, was used as the validation dataset.

**Table 1:** The detailed information of every ccRCC dataset included in the meta‐analysis.

**Accession**	**Platform**	**Number of normal samples**	**Number of cancer samples**
GSE46699	GPL570, Affymetrix U133 Plus 2.0	63	67
GSE53757	GPL570, Affymetrix U133 Plus 2.0	72	72
GSE11151	GPL570, Affymetrix U133 Plus 2.0	5	26
GSE16441	GPL6480, Agilent‐014850 Whole Human Genome Microarray	17	17
GSE36895	GPL570, Affymetrix U133 Plus 2.0	23	29
GSE66272	GPL570, Affymetrix U133 Plus 2.0	27	27
GSE71963	GPL6480, Agilent‐014850 Whole Human Genome Microarray	16	32
GSE168845	GPL21185, Agilent‐072363 SurePrint G3 Human GE v3 8x60K Microarray	4	4
GSE53000	GPL6244, Affymetrix Human Gene 1.0 ST Array	6	56
GSE76351	GPL11532, Affymetrix Human Gene 1.1 ST Array	12	12
GSE68417	GPL6244, Affymetrix Human Gene 1.0 ST Array	14	29
GSE11024	GPL6671, Affymetrix U133 Plus 2.0	12	10
GSE40435	GPL10558, Illumina HumanHT‐12 V4.0 expression beadchip	101	101

Abbreviations: GPL, gene platform; GSE, gene expression data series.

### 2.2. Analysis of DEGs

**Figure 1:**
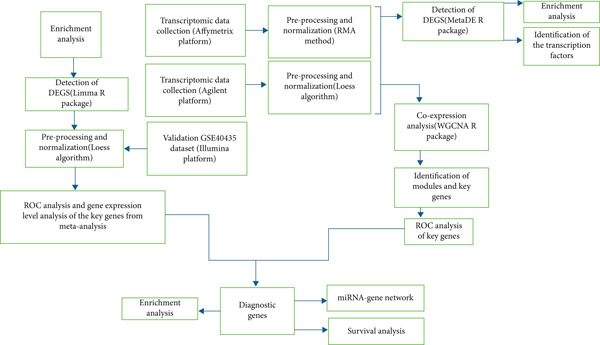
Workflow including meta‐analysis and bioinformatics pipeline.

Preprocessing was done individually for both platforms (Figure 1). Affymetrix data were normalized with the robust multiarray average (RMA) method (https://rdrr.io/bioc/affy/man/rma.html). Normalization was done for Agilent microarray data with the Loess algorithm from the limma Bioconductor package (Version 3.62.2) [6]. The Probeset IDs were converted to corresponding gene symbols utilizing the relevant probe annotation files. Where probes were mapping into the same gene symbol, the mean expression value was computed by utilizing the aggregate function. Moreover, genes with low expression levels were eliminated from the downstream analysis. After batch effect correction through the sva R package (Version 3.54.0) [7], the datasets were merged into a single dataset and analyzed through two different meta‐analytic approaches with the MetaDE package (Version 1.0.5) in R software [8], one focusing on the combination of *p* values and the other on the combination of effect sizes. Meta‐analysis of differential expressions between ccRCC and normal samples was performed by the integration of *p* values with Fisher′s method. To improve the precision of DEG identification, gene expression variation across all datasets was estimated by aggregating individual effect sizes into a unified meta‐effect size using a random effects model.

After this integration, the top genes were identified through the z‐statistic, and *p* values were rigorously corrected for multiple hypotheses testing through Benjamini–Hochberg false discovery rate (FDR) adjustment. Due to very high heterogeneity in gene expression between samples and datasets, a very conservative FDR cutoff value of <1 × 10^−19^ was employed within both integrative analytic platforms in order to provide solid and certain identification of DEGs. Raw expression data for the GSE40435 dataset were normalized using cyclic loess normalization to ensure consistency across samples. After normalization, differential expression between ccRCC and normal tissue samples was tested with the help of the limma package within R Version 3.62.2 [9]. Genes with an adj.p.val < 1 × 10^−19^ and a log_2_fold change > 0.5 were defined to be upregulated, and genes with log_2_fold change < −0.5 were taken as downregulated.

### 2.3. Functional Annotation and Pathway Enrichment Profiling

In order to detect key pathways and genes involved in ccRCC, Gene Ontology (GO) analysis including the cellular component (CC), molecular function (MF), and biological process (BP) [10] and Kyoto Encyclopedia of Genes and Genomes (KEGG) [11] pathway analysis were carried out on DEGs using the enrichGO and enrichKEGG function from the clusterProfiler R package (Version 4.14.6) [12]. DEGs were split into up and downregulated genes in the first instance and then annotated against the human genome using org.Hs.eg.db database (Version 3.20.0), where gene symbols were used as the key identifier. GO terms and KEGG pathways with a corrected *p* value < 0.05 and the Benjamini–Hochberg pAdjust method for multiple testing correction were deemed to be statistically significant enrichment. A filter on gene set size was implemented as well, with a minimum of 10 genes and a maximum of 500 genes for each GO term to eliminate very small or very big terms to ensure the results are biologically interpretable. The Top 20 most enriched GO terms and KEGG pathways in DEGs were then plotted with dot plots.

### 2.4. Identification of Transcription Factors (TFs)

The Human Transcription Factors database (http://humantfs.ccbr.utoronto.ca/allTFs.php) was used to determine TFs associated with the DEGs.

### 2.5. WGCNA

To elucidate the coexpression architecture of DEGs and discern modules of tightly correlated gene clusters, the WGCNA R package (Version 1.73) was implemented [13]. After correcting for batch effects, a similarity matrix was constructed using Pearson correlation coefficients between all pairs of DEGs, which was subsequently transformed into an adjacency matrix through a soft‐thresholding power function (*β*). The adjacency matrix was then converted into a topological overlap matrix (TOM) to enhance network connectivity interpretation. Subsequently, a hierarchical clustering dendrogram was generated, and gene modules were delineated using the cutreeDynamic function, with a predefined minimum module size threshold of 30 genes. For each identified module, protein–protein interaction (PPI) networks were analyzed using the STRING database. To identify key regulatory genes, the Top 10 hub genes within each module were prioritized based on the network centrality metrics including degree, betweenness, and maximal clique centrality (MCC), as computed by the CytoHubba plugin within Cytoscape software (Version 3.9.0).

### 2.6. Identification of Diagnostic Genes

Key genes identified through WGCNA were further evaluated using ROC curve analysis, with AUC values calculated via the *pROC* package in R (Version 1.19.0.1) [14] to assess their predictive performance. To examine expression patterns, the expression levels of these genes were visually compared between ccRCC patients and healthy controls in the validation dataset GSE40435 using boxplots. Diagnostic genes were subsequently curated from both the integrated and validation cohorts using a rigorous threshold of AUC > 0.700.

### 2.7. Survival Analysis for Identification of Prognostic Biomarkers

To determine the prognostic value of the diagnostic hub genes, we conducted a Kaplan–Meier overall survival analysis for KIRC (kidney renal clear cell carcinoma) using the Gene Expression Profiling Interactive Analysis (GEPIA3) online tool (https://gepia3.bioinfoliu.com/). We examined statistical significance with the Mantel–Cox (log‐rank) test, where a *p* value of less than 0.05 indicates a significant relationship. We also estimated hazard ratios (HRs) by applying the Cox proportional‐hazards model to measure the impact of gene expression on the survival outcomes of patients.

### 2.8. Construction of the miRNA–Gene Regulatory Network

To elucidate posttranscriptional regulatory interactions, the miRNet 2.0 database [15] was employed to predict putative interactions between the identified diagnostic genes and their corresponding microRNAs (miRNAs). The resulting miRNA–gene regulatory network was subsequently constructed and graphically rendered using the integrated visualization capabilities of miRNet. To identify the miRNAs that target hub genes, the miRTarBase database was explored (https://mirtarbase.cuhk.edu.cn/%7EmiRTarBase/miRTarBase_2025/php/index.php).

## 3. Results

### 3.1. Identification of the Most Upregulated and Downregulated Genes and Their Associated Pathways in ccRCC

For this study, DEG identification for each dataset was done separately through the GEO2R online tool. Genes with an adjusted *p* value < 0.05 and |log_2_ fold change| > 0.5 were considered significantly differentially expressed (Supporting Information 1: Figure S1A–L). Supporting Information 2: File S1 A–L represents log2 fold change and *p* value of DEGs from GSE11024, GSE11151, GSE16441, GSE36895, GSE46699, GSE53000, GSE53757, GSE66270, GSE68417, GSE71963, GSE76351, and GSE168845 datasets.

Prior to performing the meta‐analysis, principal component analysis (PCA) was performed to determine and visualize the effectiveness of batch‐effect correction. As can be seen from Supporting Information 1: Figure S2A, originally the samples had been separated based on their dataset of origin, signifying the presence of powerful batch effects, whereas upon correction with ComBat the datasets became well‐blended and moderate separation between tumor and normal samples was observed, corroborating the fact that the technical variation had been properly eliminated but the biological differences remained (Supporting Information 1: Figure S2B). Then, the MetaDE R package was employed to identify DEGs across datasets. We applied two meta‐analysis methods to identify DEGs in ccRCC. By integrating *p* values across datasets using Fisher′s sum of logs method, a total of 8177 DEGs were initially identified using FDR threshold of <1 × 10^−19^. To further enhance the specificity of DEG detection in ccRCC, a random effects meta‐analysis was performed, synthesizing individual effect sizes from each dataset into a comprehensive meta‐effect size. This approach yielded 5029 DEGs under a highly stringent FDR threshold of <1 × 10^−19^. A comparative analysis using a Venn diagram revealed 4651 overlapping DEGs, comprising 2401 upregulated and 2250 downregulated genes, common to both statistical frameworks (Supporting Information 1: Figure S3 and Supporting Information 3: Table S1). These intersecting genes represent those with both statistically significant expression changes and consistently large effect sizes across all datasets, thus reinforcing their biological relevance and robustness. We next conducted pathway enrichment analysis of the DEGs from the meta‐analysis using clusterProfiler, incorporating BP, MF, and CC terms. To reduce redundancy among enriched terms, simplify function within clusterProfiler was used. The results revealed significant enrichment of upregulated DEGs in BP category related to immune response, cell adhesion regulation, leukocyte activation, and chemotaxis (Figure 2a and Supporting Information 3: Table S2). Downregulated DEGs were mainly involved in metabolism and generation of metabolites and energy (Figure 2b and Supporting Information 3: Table S3). We identified 1082 DEGs (adj.p.val < 1 × 10^−19^ and |log_2_ FC| > 0.5) in validation GSE40435 dataset with 462 upregulated and 620 downregulated genes (Supporting Information 3: Table S4). BP enrichment analysis of upregulated DEGs displays the same outcome as that of upregulated DEGs from meta‐analysis (Figure 2c and Supporting Information 3: Table S5). Moreover, additional downregulated DEGs from the GSE40435 dataset showed common pathways with downregulated DEGs from meta‐analysis (Figure 2d and Supporting Information 3: Table S6). The GO‐CC enrichment revealed that DEGs overexpressed were most significantly connected with cell adhesion, migration, and cytoskeleton remodeling (Supporting Information 1: Figure S4A and Supporting Information 3: Table S7), whereas underexpressed DEGs were connected with cell polarity and membrane organization (Supporting Information 1: Figure S4B and Supporting Information 3: Table S8).

Figure 2GO term enrichment analysis of the BP category for upregulated and downregulated DEGs from the integrated and validation dataset. (a, b) Dot plots display the BP pathways for upregulated and downregulated DEGs from the integrated dataset (tumor vs. normal samples). (c, d) The BP pathways associated with upregulated and downregulated DEGs from the GSE40435 dataset (tumor vs. normal samples). The Top 20 enriched GO terms, ranked by gene count, are presented in each plot. The size of each dot corresponds to the number of genes associated with the GO‐BP term (pink arrows), and the color reflects the adjusted *p* value. The numbers above each dot represent the actual adjusted *p* value. The *x*‐axis represents the gene proportion within GO‐BP term relative to the total number of genes in that term, and the *y*‐axis lists the enriched GO terms from the BP category. A complete list of GO terms from the BP category for the integrated dataset can be found in Supporting Information 3: Tables S2 and S3. A complete list of GO terms from the BP category for the validation dataset can be found in Supporting Information 3: Tables S5 and S6. GO, Gene Ontology; DEGs, differentially expressed genes; BP, biological process.

**Figure:**
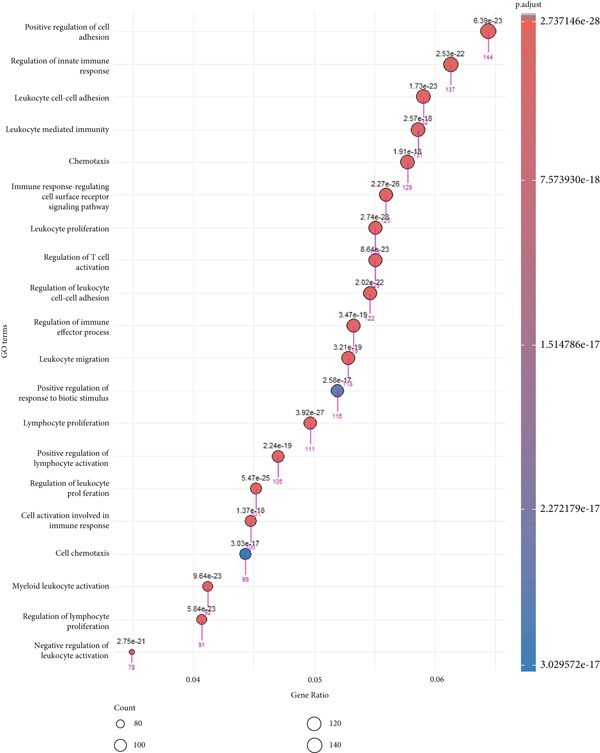
(a)

**Figure:**
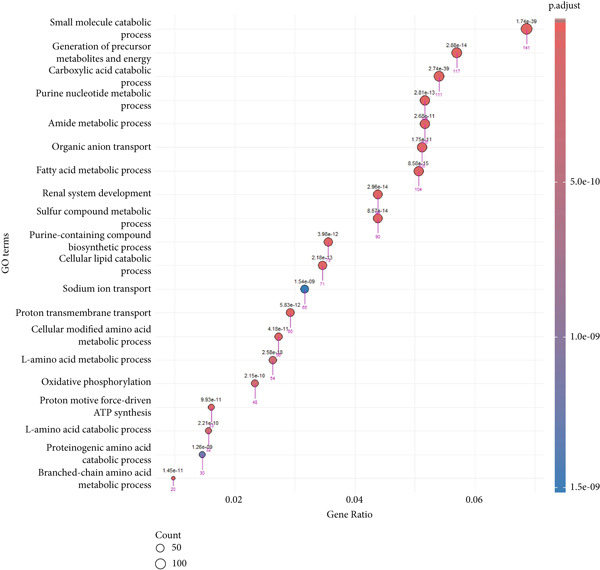
(b)

**Figure:**
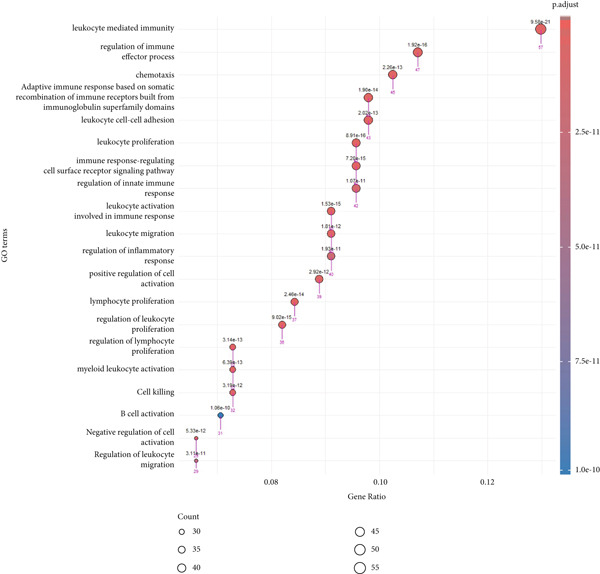
(c)

**Figure:**
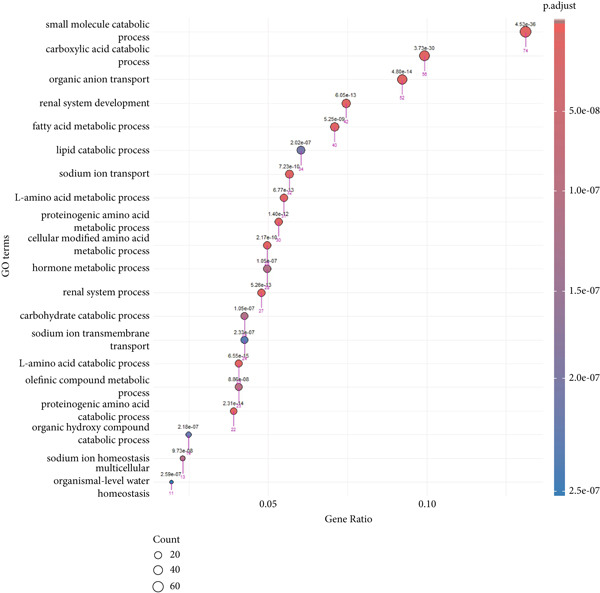
(d)

In GO‐MF analysis, the upregulated DEGs were primarily enriched in GTPase activity, acting binding, and receptor–cytokine interactions (Supporting Information 1: Figure S4C and Supporting Information 3: Table S9), while downregulated DEGs were enriched in transmembrane transporter activities and metabolic energy production (Supporting Information 1: Figure S4D and Supporting Information 3: Table S10). Validation DEGs had overlapping pathways with the integrated dataset for both GO‐CC and GO‐MF categories (Supporting Information 1: Figure S4E–H and Supporting Information 3: Tables S11, S12, S13, and S14). KEGG pathway enrichment analysis found that the upregulated DEGs in the merged dataset were enriched in immune system and microbial defense pathways (Supporting Information 1: Figure S5A and Supporting Information 3: Table S15), while downregulated DEGs were primarily associated with amino acid metabolism (Supporting Information 1: Figure S5B and Supporting Information 3: Table S16). Validation dataset upregulated DEGs shared similar pathways (Supporting Information 1: Figure S5C and Supporting Information 3: Table S17), and validation dataset downregulated DEGs shared amino acid metabolism as a significantly enriched pathway (Supporting Information 1: Figure S5D and Supporting Information 3: Table S18).

### 3.2. Identification of the TFs

**Figure 3:**
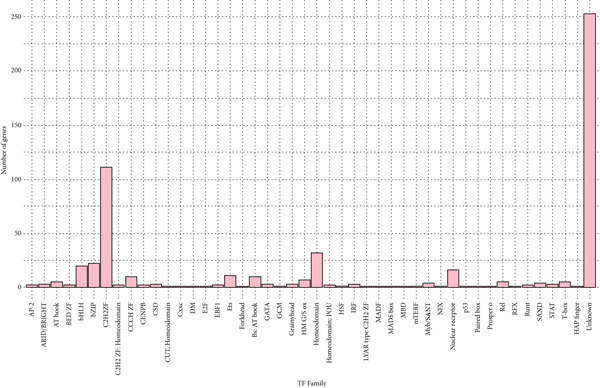
Number of differentially expressed genes (DEGs) in each TF family.

Among the DEGs, a total of 291 TFs were identified, spanning 46 evolutionarily conserved TF families. Notably, two families emerged as predominant: an unclassified group comprising 253 TFs and the C2H2 zinc finger (ZF) family, encompassing 113 TFs (Figure 3 and Supporting Information 3: Table S19).

### 3.3. WGCNA and Identification of Module Central Genes

The WGCNA framework was implemented to dissect gene coexpression architectures within the mRNA expression landscape of ccRCC. Module detection was performed using a dynamic tree cutting algorithm, with a soft‐thresholding power of *β* = 19 (Figure 4a, left and right plots) and a minimum module size set at 30 genes. This analysis delineated eight distinct gene coexpression modules, each assigned a unique color label (Figure 4b). Module sizes ranged from 46 genes in the pink module to 418 genes in the turquoise module (Supporting Information 3: Table S20). To identify key regulatory elements within each module, we utilized the CytoHubba plugin in Cytoscape to prioritize hub genes based on network centrality. The Top 10 hub genes within each module were determined using three centrality metrics: MCC, betweenness, and degree, thereby highlighting genes with prominent topological importance in the coexpression network. By comparing the results from all three filters, we found the common genes that were consistently identified across all three algorithms. Ultimately, we identified a total of 21 genes across all modules using this approach including the following: FCGR1A, GZMB, CD163, CCL4, SLC12A1, IRF7, SUCLG1, SLC22A8, CDK1, CCNB1, AURKA, KIF11, NUF2, CCNA2, ACADM, ALDH2, ACO2, PTPRC, ITGB2, TLR4, and SPI1. Among the identified modules, the turquoise module harbored the greatest enrichment of TFs, encompassing 46 TFs, thereby underscoring its putative central regulatory function (Supporting Information 3: Table S21).

Figure 4(a) Determination of soft‐thresholding power in weighted gene coexpression network analysis (WGCNA). The left plot shows the scale‐free fit index versus soft‐thresholding power, while the right plot displays mean connectivity versus soft‐thresholding power. (b) Clustering dendrogram and modules identified through WGCNA. The dendrogram represents gene clustering based on the TOM dissimilarity measure, with each line corresponding to a single gene. The color bar beneath the dendrogram indicates the modules detected with a minimum module size of 30, resulting in eight distinct color‐coded modules.

**Figure:**
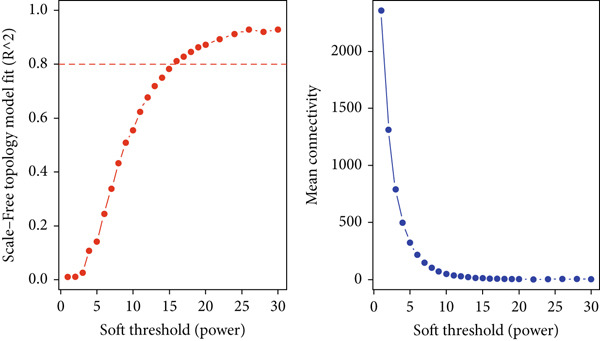
(a)

**Figure:**
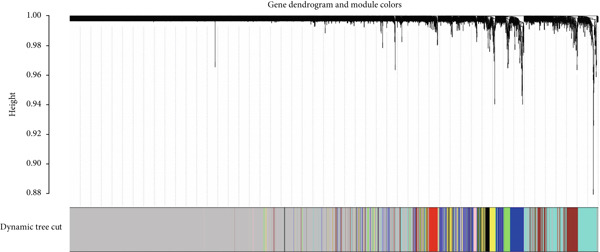
(b)

### 3.4. Discovery and Validation of Candidate Diagnostic Biomarkers

**Figure 5:**
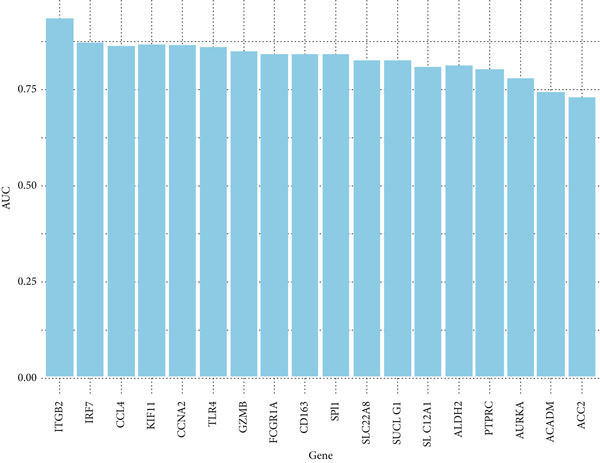
Bar plot of AUC values for genes in ROC analysis. The bar plot illustrates the diagnostic performance of 18 genes based on their AUC values, summarizing the results from the ROC analysis. Higher AUC values indicate better diagnostic accuracy.

**Figure 6:**
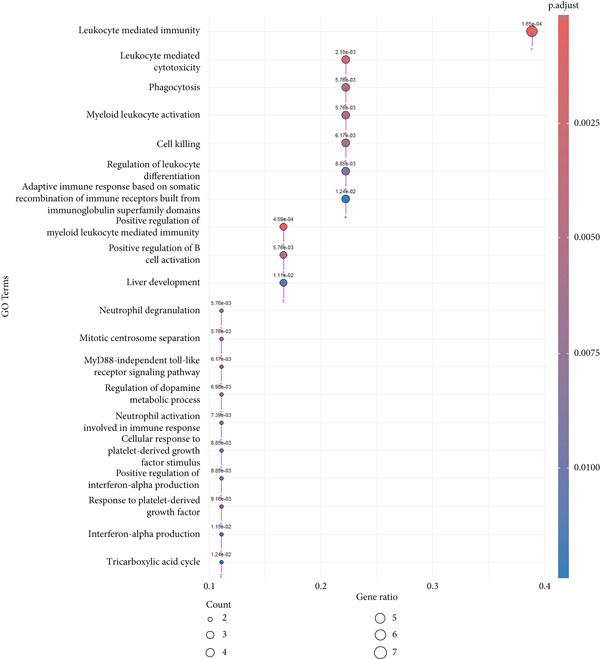
The GO‐BP term enrichment analysis of 17 diagnostic hub genes. The Top 20 enriched GO‐BP terms, ranked by gene count, are presented in each plot. The size of each dot corresponds to the number of genes associated with the GO‐BP term (pink arrows), and the color reflects the adjusted *p* value. The numbers above each dot represent the actual adjusted *p* value. The *x*‐axis represents the gene proportion within GO‐BP term relative to the total number of genes in that term, and the *y*‐axis lists the enriched GO terms from the BP category. A complete list of GO‐BP terms for the integrated dataset can be found in Supporting Information 3: Table S25. GO, gene ontology; BP, biological process.

A total of 12 microarray datasets incorporated in the meta‐analysis were integrated. Following normalization and batch effect adjustment, receiver operating characteristic (ROC) curve analysis was conducted to assess the diagnostic efficacy of 21 hub genes in ccRCC. Genes demonstrating an area under the curve (AUC) exceeding 0.700 were designated as diagnostic biomarkers (Supporting Information 3: Table S22). The results demonstrated that the AUC values for 18 hub genes exceeded 0.700, indicating robust diagnostic performance. Figure 5 depicts the diagnostic efficacy of these 18 hub genes within the integrated dataset, with ITGB2 exhibiting the highest diagnostic accuracy (AUC = 0.933), followed by IRF7 (AUC = 0.87). Furthermore, both gene expression levels and diagnostic capabilities were corroborated in an independent validation cohort. Supporting Information 1: Figure S6 illustrates the expression profiles of 21 pivotal genes within the GSE40435 validation dataset. All genes, except for CCL4, show statistically significant differences between normal and tumor samples (adjusted *p* value < 0.05) (Supporting Information 3: Table S23). Except CCL4, the AUCs of the genes were greater than 0.800 (Supporting Information 3: Table S24). Furthermore, NUF2, CCNB1, and CDK1 demonstrated AUC values greater than 0.8, whereas their AUC values in the combined dataset were 0.68, 0.69, and 0.64, respectively. Finally, we identified ALDH2, ACADM, KIF11, SPI1, IRF7, GZMB, ACO2, AURKA, CCNA2, CD163, FCGR1A, ITGB2, PTPRC, SLC12A1, SLC22A8, SUCLG1, and TLR4 as diagnostic hub genes, based on their significant differential expression and AUC values greater than 0.7 in both the combined dataset and the validation dataset. GO enrichment analysis of the 17 diagnostic hub genes revealed their significant enrichment in immune‐related biological processes, notably leukocyte‐mediated immunity, activation of myeloid leukocytes, and regulation of immune effector functions. They were also enriched in neutrophil degranulation, phagocytosis, B cell activation, centrosome separation, and MyD88‐independent toll‐like receptor signaling. Additionally, the genes were involved in dopamine and catecholamine metabolism regulation and neutrophil activation in immune responses (Figure 6 and Supporting Information 3: Table S25).

### 3.5. Survival Analysis of Hub Genes

In terms of survival analysis, we performed the analyses of diagnostic hub genes using the GEPIA3 platform (https://gepia3.bioinfoliu.com/survanl/uni/), and the HRs with 95% confidence intervals (CIs) for the significant genes are as follows: ACADM (*p* value = 3.31e − 09), HR = 0.39 (95% CI: 0.28~0.54) (Figure 7a); ACO2 (*p* value = 3.02e − 06), HR = 0.48 (95% CI: 0.35~0.66) (Figure 7b); ALDH2 (*p* value = 2.71e − 08), HR = 0.42 (95% CI: 0.31~0.58) (Figure 7c); CCNA2 (*p* value = 0.000457), HR = 1.72 (95% CI: 1.27~2.34) (Figure 7d); FCGR1A (*p* value = 5.19e − 05), HR = 1.88 (95% CI: 1.38~2.57) (Figure 7e); IRF7 (*p* value = 0.00121), HR = 1.64 (95% CI: 1.21~2.23) (Figure 7f); SLC22A8 (*p* value = 2.35e − 07), HR = 0.44 (95% CI: 0.32~0.61) (Figure 7g); SUCLG1 (*p* value = 3.17e − 05), HR = 0.52 (95% CI: 0.38~0.71) (Figure 7h); and TLR4 (*p* value = 4.52e − 05), HR = 0.53 (95% CI: 0.39~0.72) (Figure 7i) were the only genes with significant overall survival (*p* value < 0.05).

Figure 7Survival analysis of diagnostic hub genes of (a) ACADM, (b) ACO2, (c) ALDH2, (d) CCNA2, (e) FCGR1A, (f) IRF7, (g) SLC22A8, (h) SUCLG1, and (i) TLR4 based on GEPIA3 data.

**Figure:**
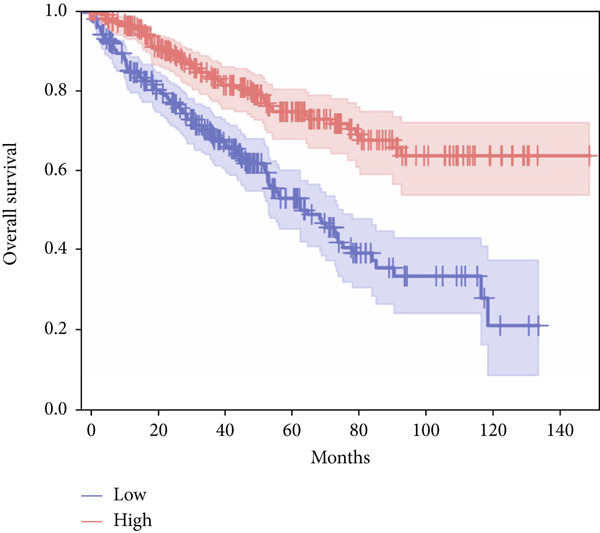
(a)

**Figure:**
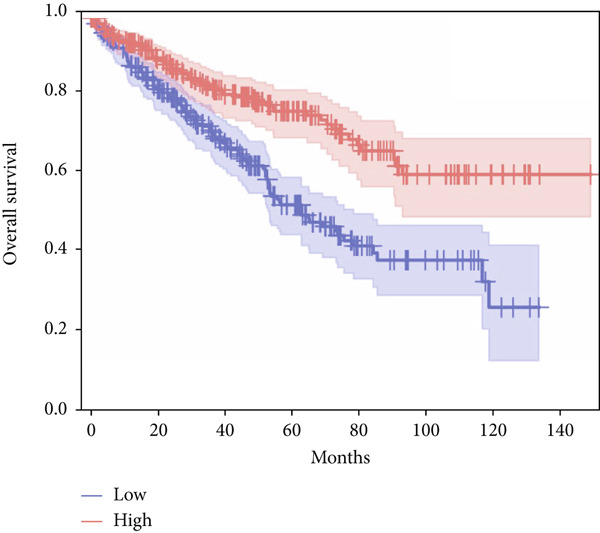
(b)

**Figure:**
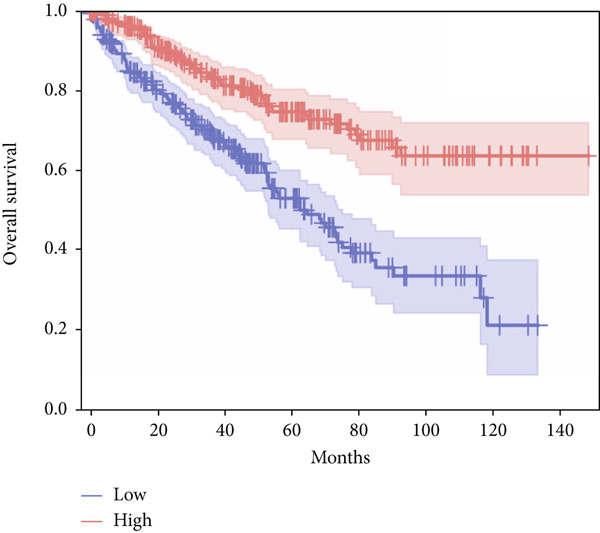
(c)

**Figure:**
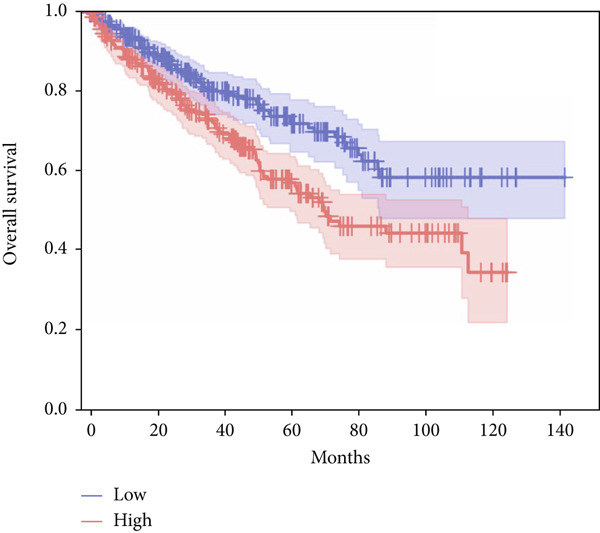
(d)

**Figure:**
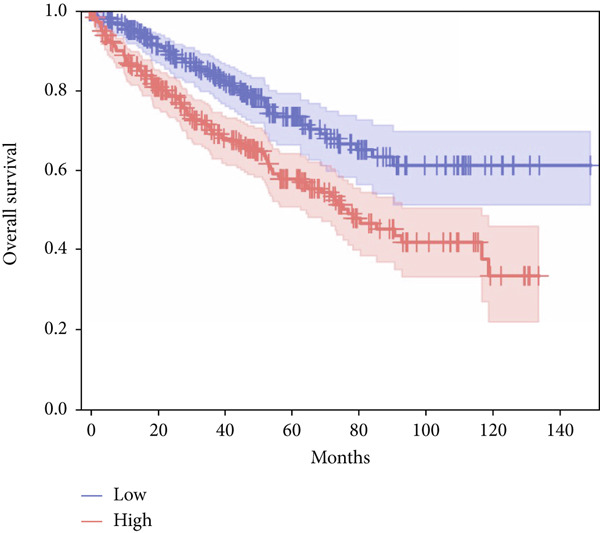
(e)

**Figure:**
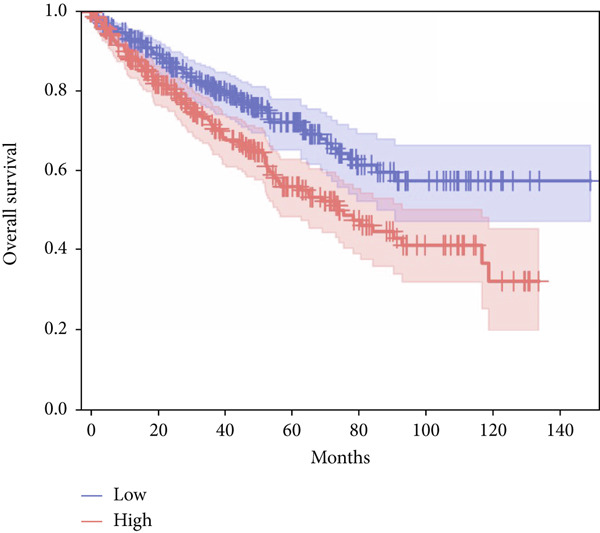
(f)

**Figure:**
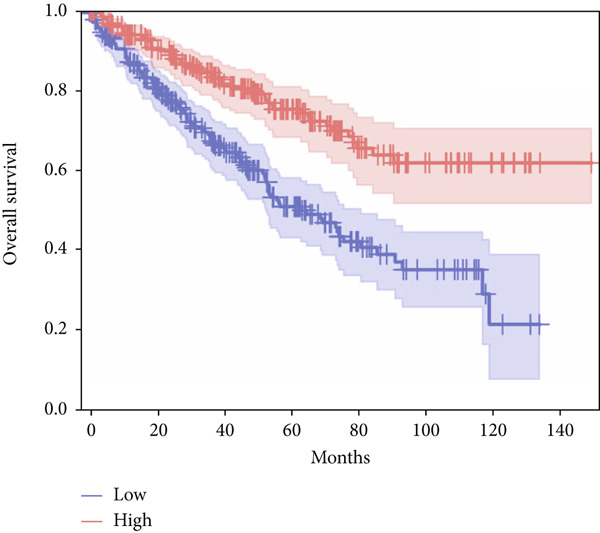
(g)

**Figure:**
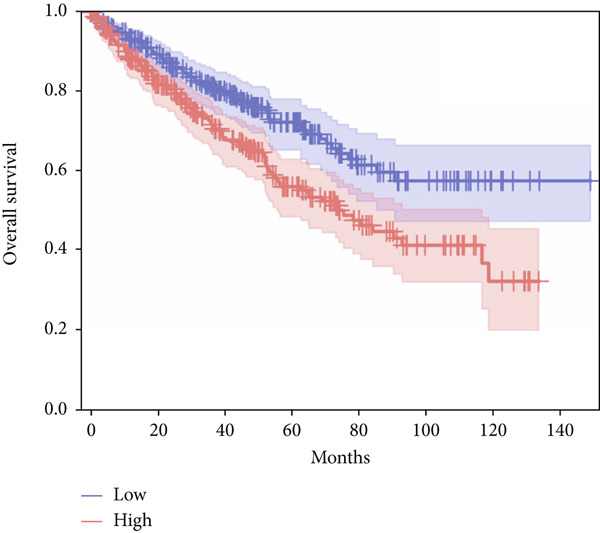
(h)

**Figure:**
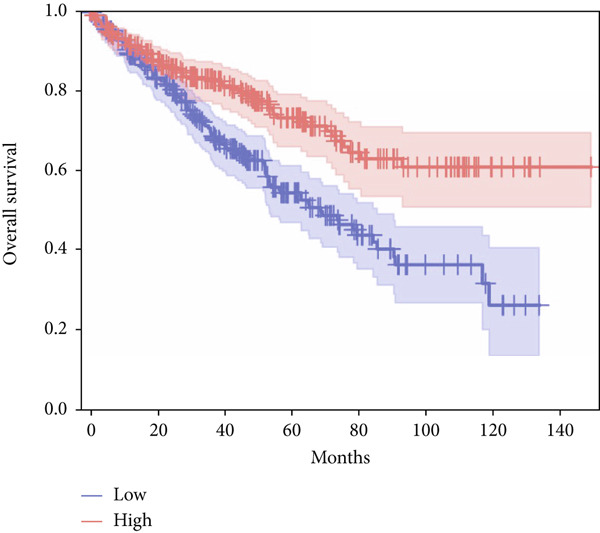
(i)

### 3.6. Prediction of Potential miRNA–Target Regulatory Networks

**Figure 8:**
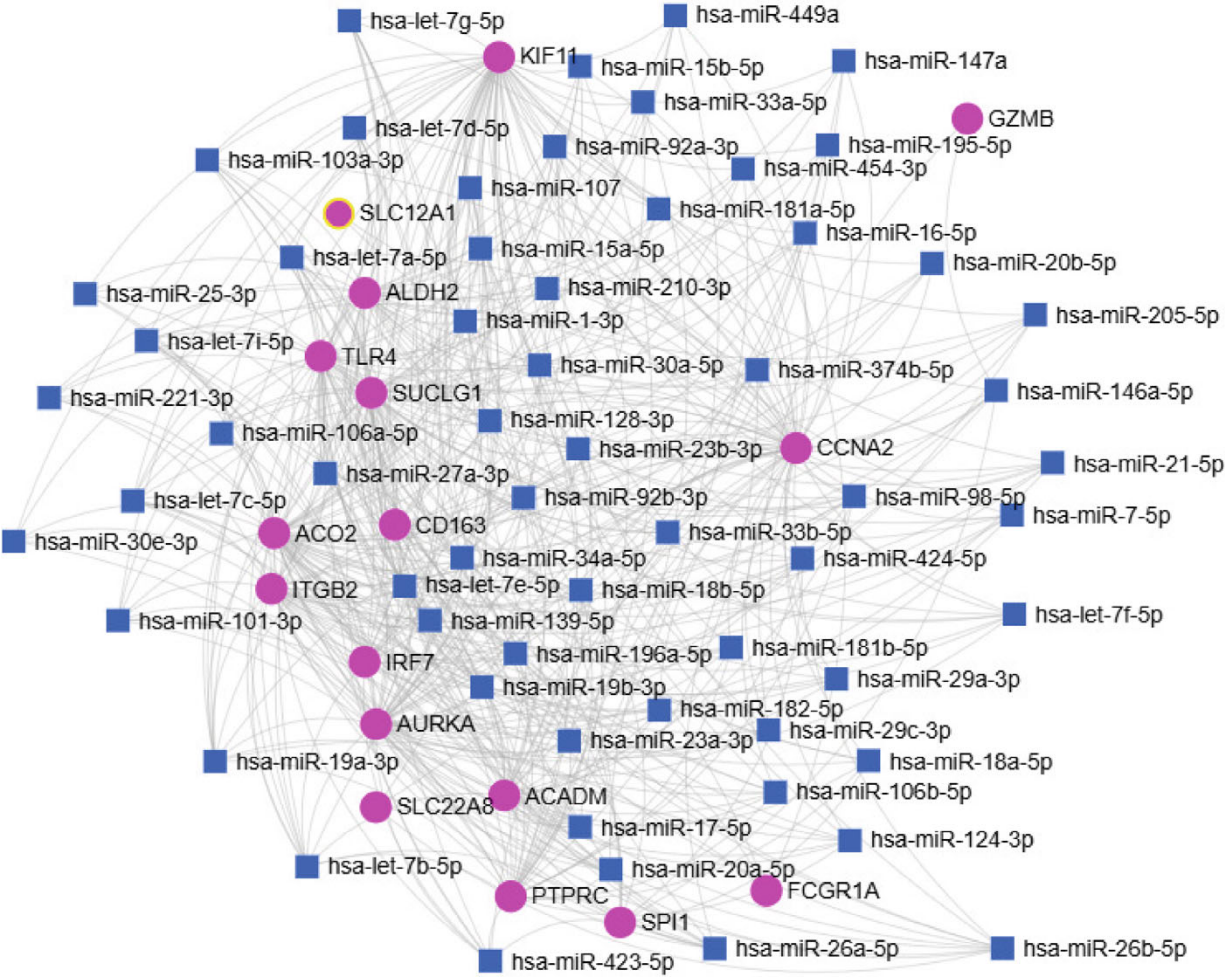
miRNA–mRNA network of 17 diagnostic hub genes by miRNet. The pink spheres represent 17 diagnostic hub genes; the blue squares represent the miRNA associated with the 17 diagnostic hub genes.

To gain a broader understanding of the functions of the diagnostic hub genes, a miRNA–mRNA regulatory network was constructed using the miRNet 2.0 database. The resulting miRNA–mRNA regulatory network was visualized (Figure 8), focusing on miRNAs with a degree of 6 or higher. According to the miRNet analysis, the miRNAs listed in Supporting Information 3: Table S26 demonstrated significant interactions with the majority of hub genes implicated in the initiation and progression of ccRCC. Notably, hsa‐let‐7b‐5p, hsa‐miR‐19a‐3p, hsa‐miR‐19b‐3p, and hsa‐miR‐34a‐5p exhibited the highest degree (12), suggesting their potential critical role in the pathogenesis and development of ccRCC. The upstream miRNAs of the hub genes investigated by miRTarBase are as follows: hsa‐let‐7b‐5p targets AURKA, CCNA2, and TLR4; hsa‐miR‐19a‐3p and hsa‐miR‐19b‐3p target CCNA2; and hsa‐miR‐34a‐5p targets KIF11.

## 4. Discussion

Over the past decades, ccRCC studies have reported a plethora of candidate biomarkers in their development. There remains a big gap, however, in the overall validation of these biomarkers in independent datasets, a necessary step to determine their clinical utility and reproducibility. This study bridges this gap by integrating a powerful meta‐analysis with independent dataset validation, demonstrating the necessity of checking consistency and diagnostic utility by rigorous validation strategies for biomarker translation.

Utilizing 652 microarray samples from various GEO datasets, our meta‐analysis identified 4651 DEGs distinguishing tumor from normal tissues. The results revealed significant enrichment of upregulated DEGs in the BP category, particularly in immune response, cell adhesion regulation, leukocyte activation, and chemotaxis. All of these pathways have been previously confirmed in the context of ccRCC [16, 17]. Downregulated DEGs were primarily involved in metabolism and the generation of metabolites and energy consistent with previous findings, suggesting enhanced metabolic deregulation in ccRCC [18–20]. BP enrichment analysis of the up and downregulated DEGs in the validation dataset showed similar results to those observed in the meta‐analysis. The CC of the GO analysis revealed the majority of enriched categories were relevant to cell adhesion, migration, and cytoskeleton remodeling, while downregulated DEGs were linked to cell polarity and membrane organization [21–24]. In addition, the results of GO MF enrichment analysis showed that upregulated DEGs were mainly enriched in GTPase activity, actin binding, and receptor–cytokine interactions, while downregulated DEGs were enriched in transmembrane transporter activities and metabolic energy production [25–28]. Validation of DEGs revealed that their pathways were consistent with those of the integrated dataset under GO‐CC and GO‐MF categories. KEGG pathway enrichment analysis revealed that the upregulated DEGs in the integrated dataset were exceedingly enriched in immune system and microbial defense pathways. Downregulated DEGs were mostly related to amino acid metabolism, as reported in earlier studies [29, 30]. The validation set also showed upregulated DEGs in similar pathways, and downregulated DEGs again pointed to amino acid metabolism as one of the top enriched pathways. This consistency reinforces the strength of our results and emphasizes the biological importance of disrupted metabolic and immune pathways in ccRCC. Notably, many of the biological processes highlighted in the GO and KEGG analyses, including immune activation, cell adhesion, and metabolic reprogramming, were directly reflected in the functions of the 17 diagnostic hub genes identified later. For example, immune activation was represented by genes such as FCGR1A, GZMB, IRF7, PTPRC, SPI1, and TLR4, all of which are involved in immune cell recruitment, activation, and immune checkpoint regulation within the tumor microenvironment [16, 31–37]. All these genes are overexpressed in ccRCC, suggesting their role in immune activation within the tumor microenvironment. FCGR1A, playing a role in antibody‐dependent immunity, is correlated with tumoral progression and prognosis. Consistent with our findings, increased expression of FCGR1A in ccRCC has been associated with worse survival outcomes [31, 32].

Similarly, GZMB, a key protease of cytotoxic T cells and NK cells, is elevated in ccRCC [33, 38, 39]. However, despite its increased expression, our findings did not reveal a significant correlation with survival.

IRF7, a master regulator of interferon signaling, correlates with advanced tumor stages and poor prognosis [34, 40]. Our data reveal that decreased IRF7 expression is linked to better patient outcomes. PTPRC, an important regulator of intracellular signaling, is also overexpressed in ccRCC [16, 41]. In our analysis, however, it failed to show a strong correlation with survival. SPI1, encoding the TF PU.1, is involved in immune cell differentiation and has been implicated in immune cell infiltration in ccRCC [36, 37, 42, 43], although similar to GZMB and PTPRC, it was not significantly correlated with survival in our analysis.

Lastly, TLR4, a pattern recognition receptor, is an independent predictor of prognosis, promoting tumor cell proliferation, migration, and immune modulation [35]. TLR4 overexpression in ccRCC was related to improved survival [44], suggesting that immune stimulation through TLR4 signaling may play a protective role on ccRCC development.

Cell adhesion was prominently reflected in genes like ITGB2 and CD163 that play a key role in cell–cell and cell–matrix interactions [21, 45, 46]. TGB2, which is a subunit of integrin involved in cell adhesion, and CD163, which is predominantly expressed in monocytes and macrophages, overexpression was correlated with tumor aggressiveness and poor survival in RCC [16, 47, 48]. In our analysis, however, the statistical significance of these associations remains limited.

Metabolic reprogramming, another key process highlighted in the GO and KEGG analyses, was directly linked to genes like ACO2, SUCLG1, ALDH2, ACADM, SLC12A1, and SLC22A8. ALDH2, ACADM, ACO2, SUCLG1, SLC22A8, and SLC12A1 are all downregulated in ccRCC compared to normal tissues, which suggests key disruptions in metabolic regulation and cellular processes leading to tumor formation. In ccRCC, reduced ALDH2 expression has been associated with dysregulation of detoxification and stress response mechanisms. This disruption in cellular protection leads to an increased accumulation of toxic aldehydes, which may enhance tumorigenesis and ccRCC progression [49–51]. Similarly, the downregulation of ACADM, a key enzyme involved in fatty acid oxidation, is strongly linked to advanced tumor stage, higher histological grade, and poorer overall and disease‐free survival and serves as an independent prognostic indicator [52]. Our findings resonate powerfully with prior reports. The expression of ALDH2 and ACADM was found to be reduced in RCC tissues compared to matched adjacent normal tissues, and they exhibited a protective effect in renal clear cell carcinoma. Reduced levels of ACO2, a mitochondrial Fe–S enzyme catalyzing citrate isomerization in the Krebs cycle, SUCLG1, a rate‐limiting enzyme in the tricarboxylic acid cycle, and SLC22A8, mediating the secretion of endogenous and exogenous anions, are significantly associated with poorer overall survival in RCC patients [53–55], aligning with our findings.

AURKA, CCNA2, and KIF11 are all key regulators of the cell cycle. These genes are overexpressed in ccRCC and by disrupting the cell cycle contribute to tumor progression. AURKA, a critical kinase that orchestrates mitosis, activates CCNB1 and results in uncontrolled cell growth [56], while KIF11 is central to spindle formation and is implicated in tumor aggressiveness [57]. CCNA2, which regulates the G1/S and G2/M checkpoints, accelerates cell cycle progression and enhances tumor growth [58, 59]. However, in our analysis, only CCNA2 was statistically significantly associated with survival, linking its overexpression to poorer prognosis.

The lack of prognostic significance for 8 of the 17 ccRCC hub genes may be due to several biological reasons. Among the prominent ones is tumor heterogeneity, because gene expression varies significantly across the different subtypes and stages of ccRCC [60], and therefore, some genes may not show consistent associations with survival. Further, functional redundancy in signaling pathways can also decrease the prognostic potential of certain genes, especially if other genes compensate for their loss [61]. The tumor microenvironment also plays an important part; infiltration by immune cells, stromal interaction, and vascularization can all impact gene expression and thereby change the prognostic potential of certain genes [62]. Also, posttranscriptional regulation can generate discordance between mRNA levels and functional protein activity, leading to the lack of survival association in the context of high gene expression [63].

To better understand the regulatory mechanisms driving ccRCC, we conducted a TF analysis, which identified 113 TFs among the DEGs, with the C2H2‐ZF family being the most prominent. These versatile regulators control vast gene networks involved in disease progression. Somatic mutations in C2H2‐ZF members are known to contribute to widespread transcriptional dysregulation in cancers [64, 65]. In the present study, several TFs from the C2H2‐ZF family were identified as being involved in the tumorigenesis of ccRCC. Among them, ZNF692 promotes tumor proliferation by repressing essential genes [66]. ZNF395 enhances proliferation and invasion under VHL deficiency [67, 68] and ZNF582 is frequently hypermethylated and downregulated, correlating with worse prognosis [69].

Additionally, the miRNA–gene regulatory network highlights potential posttranscriptional regulators of these diagnostic hub genes, further complicating their regulatory mechanisms. Notably, our findings show that hsa‐let‐7b‐5p, hsa‐miR‐19a‐3p, hsa‐miR‐19b‐3p, and hsa‐miR‐34a‐5p have the highest degree, suggesting their critical role in the pathogenesis and development of ccRCC. Previous studies have linked several of these miRNAs to ccRCC [70, 71].

hsa‐let‐7b‐5p, known for its tumor‐suppressive properties within ceRNA networks, has been shown to target AURKA [72, 73], CCNA2, and TLR4. By modulating AURKA and CCNA2 levels, hsa‐let‐7b‐5p influences key cellular processes such as the cell cycle, apoptosis, and mitotic progression [74]. This highlights the need for further investigation into the interaction between AURKA,CCNA2, and hsa‐let‐7b‐5p in ccRCC, with potential as a therapeutic agent or biomarker. Its regulation of TLR4 suggests a role in activating NF‐*κ*B and modulating downstream genes involved in inflammation and immune responses in ccRCC [75].

hsa‐miR‐34a‐5p, another key miRNA identified in RCC tumorigenesis and progression [71, 76], targets KIF11 [77]. By targeting KIF11, hsa‐miR‐34a‐5p may suppress abnormal mitotic activity and prevent tumorigenesis [78]. The ability of hsa‐miR‐34a‐5p to regulate such critical mitotic proteins positions it as a key tumor suppressor miRNA in ccRCC, potentially offering a therapeutic strategy for limiting tumor progression.

Additionally, the miRNAs hsa‐miR‐19a‐3p and hsa‐miR‐19b‐3p, part of the miR‐17‐92 cluster, target CCNA2 [79], reinforcing the role of this cyclin in cell cycle regulation and tumor progression. These miRNAs are known to promote tumorigenesis by enhancing the proliferation of cancer cells and have been implicated in poor prognosis across multiple cancers [80–83]. By regulating CCNA2, hsa‐miR‐19a‐3p and hsa‐miR‐19b‐3p might contribute to the dysregulation of cell cycle checkpoints, facilitating uncontrolled cell division and contributing to the aggressive nature of ccRCC. In summary, the regulatory roles of these miRNAs provide novel insights into the molecular mechanisms underlying ccRCC, highlighting their potential as therapeutic targets for future treatment strategies.

Although many of the hub genes identified have been reported in previous studies, the novelty of this work lies in its comprehensive and systematic approach to identifying and validating biomarkers for ccRCC. This study integrates a large‐scale meta‐analysis of multiple microarray datasets, offering a more robust and reliable identification of biomarkers compared to previous studies that often relied on smaller or less diverse datasets. By employing WGCNA on the meta‐analyzed DEGs, we not only identified key regulatory modules but also incorporated rigorous selection criteria based on MCC, betweenness, and degree to ensure that the regulatory elements identified are biologically relevant and critical to ccRCC pathogenesis. The validation of our findings with an independent dataset of 101 normal and 101 tumor samples adds a significant layer of confidence in the robustness of our results. Furthermore, the diagnostic value of the 21 hub genes identified was confirmed through rigorous ROC curve analysis, highlighting their potential as reliable diagnostic biomarkers. To strengthen clinical relevance, we also performed survival analysis on the 17 common hub genes identified between the integrated and validation datasets, revealing their significant prognostic potential.

This multifaceted approach, combining bioinformatics analysis with rigorous validation, is a key strength of this study. It not only identifies a set of biomarkers with superior diagnostic and prognostic potential but also offers a new, diagnostic panel for ccRCC that has clinical implications. The gene panel proposed by us consisting of 17 diagnostic hub genes might offer great potential in the early diagnosis and prognosis of renal cancer. These results show that, if confirmed in larger and more appropriate cohorts, this panel of genes may potentially be used as biomarkers for noninvasive diagnostic tests, such as blood or urine tests. However, further clinical validation is required before inclusion of these genes into diagnostic purposes. Additionally, being involved in important molecular pathways of kidney carcinogenesis, these genes could even have potential as targets for tailored therapy approaches.

One limitation of this study is that it only utilizes microarray datasets, which, while foundational, do not offer the comprehensive coverage that RNA sequencing provides. It must also be noted that mRNA expression is not necessarily a reflection of protein abundance or biological activity. Therefore, while we discuss key hub genes, their clinical relevance remains uncertain without experimental confirmation at the protein level. We note that further validation at the protein level is necessary before these genes can be progressed to the clinic. Another limitation is the use of the TCGA‐KIRC dataset for survival analysis, which is specifically aimed at ccRCC. While this dataset is helpful in providing insight into ccRCC survival, it may not be representative of all patient cohorts or subtypes in the broader ccRCC population. Consequently, the findings of this research might not be completely generalizable to all ccRCC patient populations. Further studies based on more diverse datasets or additional cohorts would be worthwhile replicating these findings in different patient populations. While our study utilized the STRING and miRNet 2.0 databases to construct PPI and miRNA–mRNA regulatory networks, respectively, it must be mentioned that these are computational predictions from curated databases and that these analyses are based on computational predictions derived from curated databases. Both predicted and experimentally validated interactions are included in these databases, which may not accurately represent the exact biological context of the ccRCC samples analyzed in this study. In addition, the interactions herein reported may be influenced by the inherent limitations and biases of the prediction algorithms and data sources utilized. Thus, while these analyses are insightful, experimental confirmation is required to determine the biological relevance of the interactions discovered in ccRCC.

## 5. Conclusion

In conclusion, this study validated 17 diagnostic hub genes through integrative bioinformatics analysis and independent dataset confirmation, reinforcing their critical roles in the pathogenesis of ccRCC. The consistent association of these genes with key pathways involved in ccRCC pathogenesis highlights their biological significance and potential utility as biomarkers. Importantly, the validation of multiple biomarkers supports the feasibility of developing a multigene panel, which holds promise for enhancing diagnostic accuracy and prognostic assessment in ccRCC. Such multigene biomarkers can improve disease stratification and guide personalized treatment strategies. While this study is fully computational, future work will include experimental validation using techniques such as qPCR and IHC to confirm the biological relevance of the identified hub genes in ccRCC. Overall, this study strengthens the foundation for biomarker‐driven precision medicine aimed at improving outcomes for ccRCC patients.

## Ethics Statement

The authors have nothing to report.

## Conflicts of Interest

The authors declare no conflicts of interest.

## Author Contributions

**Haideh Namdari:** writing – original draft, investigation, formal analysis, methodology, data curation, supervision, writing – review and editing of the manuscript. **Farhad Rezaei:** validation, conceptualization, writing – review and editing of the manuscript. **Maryam Karamigolbaghi:** writing – review and editing of the manuscript.

## Funding

No funding was received for this manuscript.

## Supporting Information

Additional supporting information can be found online in the Supporting Information section.

## Supporting information


**Supporting Information 1** Figure S1. Volcano plots depicting differential gene expression (DEG) analysis results for each dataset, performed independently using the GEO2R online tool. Figure S2. Principal component analysis (PCA) before and after batch‐effect correction. Figure S3. Venn diagram showing the overlap of differentially expressed genes (DEGs) identified by combining *p* values using Fisher′s sum of logs method and by combining individual effect sizes using a random effects model. Figure S4. (A) This figure shows cellular component enrichment analysis of upregulated DEGs in integrated dataset. (B) This figure shows cellular component enrichment analysis of downregulated DEGs in integrated dataset. (C) This figure shows molecular function enrichment analysis of upregulated DEGs in integrated dataset. (D) This figure shows molecular function enrichment analysis of downregulated DEGs in integrated dataset. (E) This figure shows cellular component enrichment analysis of upregulated DEGs in the GSE40435 dataset. (F) This figure shows cellular component enrichment analysis of downregulated DEGs in the GSE40435 dataset. (G) This figure shows molecular function enrichment analysis of upregulated DEGs in the GSE40435 dataset. (H) This figure shows molecular function enrichment analysis of downregulated DEGs in the GSE40435 dataset. Figure S5 (A) This figure shows KEGG pathway enrichment analysis of upregulated DEGs in integrated dataset. (B) This figure shows KEGG pathway enrichment analysis of downregulated DEGs in integrated dataset. (C) This figure shows KEGG pathway enrichment analysis of upregulated DEGs in the GSE40435 dataset. (D) This figure shows KEGG pathway enrichment analysis of downregulated DEGs in the GSE40435 dataset.

**Supporting Information 2** File S1 (A–L) Represent log2 (fold change) and *p* value of DEGs from GSE11024, GSE11151, GSE16441, GSE36895, GSE46699, GSE53000, GSE53757, GSE66270, GSE68417, GSE71963, GSE76351, and GSE168845 datasets.

**Supporting Information 3** Table S1. Shared DEGs identified by both statistical frameworks via Venn diagram analysis. Table S2. BP enrichment analysis of upregulated DEGs form integrated dataset using clusterProfiler. Table S3. BP enrichment analysis of downregulated DEGs form integrated dataset using clusterProfiler. Table S4. List of DEGs in the validation GSE40435 dataset. Table S5. BP enrichment analysis of upregulated DEGs in the validation GSE40435 dataset. Table S6. Biological process enrichment analysis of downregulated DEGs in the validation GSE40435 dataset. Table S7. Cellular component enrichment analysis of upregulated DEGs in integrated dataset. Table S8. Cellular component enrichment analysis of downregulated DEGs in integrated dataset. Table S9. Molecular function enrichment analysis of upregulated DEGs in integrated dataset. Table S10. Molecular function enrichment analysis of downregulated DEGs in integrated dataset. Table S11. Cellular component enrichment analysis of upregulated DEGs in the GSE40435 dataset. Table S12. Cellular component enrichment analysis of downregulated DEGs in the GSE40435 dataset. Table S13. Molecular function enrichment analysis of upregulated DEGs in the GSE40435 dataset. Table S14. Molecular function enrichment analysis of downregulated DEGs in the GSE40435 dataset. Table S15. KEGG pathway enrichment analysis of upregulated DEGs in the integrated dataset. Table S16. KEGG pathway enrichment analysis of downregulated DEGs in the integrated dataset. Table S17. KEGG pathway enrichment analysis of upregulated DEGs in the GSE40435 dataset. Table S18. KEGG pathway enrichment analysis of downregulated DEGs in the GSE40435 dataset. Table S19. Summary of transcription factors (TFs) identified among DEGs. Table S20. List of eight gene coexpression modules identified by dynamic tree cutting (*β* = 19, minimum size = 30 genes). Table S21. Transcription factor enrichment in the turquoise gene coexpression module. Table S22. Diagnostic biomarkers identified by ROC analysis of 21 hub genes in the integrated dataset. Table S23. Expression profiles of 21 pivotal genes among DEGs in the GSE40435 validation dataset. Table S24. Diagnostic performance of genes in the GSE40435 validation set. Table S25. GO‐BP enrichment analysis of 17 diagnostic hub genes. Table S26. List of miRNAs included in the miRNA–mRNA regulatory network, limited to those with a degree of 6 or higher.

## Data Availability

The datasets used in this study are accessible in the GEO database (https://www.ncbi.nlm.nih.gov/geo/) and are cited in the article. Other data presented in this study are provided in the article and its supporting information. Any additional data not included in the article are available upon request from the corresponding author.
