# Supplementary material for: Integrative Meta‐Analysis and WGCNA Reveal Candidate Diagnostic Hub Genes in Clear Cell Carcinoma
Source: Int J Cell Biol. 2026 Feb 2;2026:5567255. doi: 10.1155/ijcb/5567255 (PMC12865128; doi:10.1155/ijcb/5567255)
Supplement: Supplementary file 1 — Supporting Information 1 Figure S1. Volcano plots depicting differential gene expression (DEG) analysis results for each dataset, performed independently using the GEO2R online tool. Figure S2. Principal component analysis (PCA) before and after batch‐effect correction. Figure S3. Venn diagram showing the overlap of differentially expressed genes (DEGs) identified by combining p values using Fisher′s sum of logs method and by combining individual effect sizes using a random effects model. Figure S4. (A) This figure shows cellular component enrichment analysis of upregulated DEGs in integrated dataset. (B) This figure shows cellular component enrichment analysis of downregulated DEGs in integrated dataset. (C) This figure shows molecular function enrichment analysis of upregulated DEGs in integrated dataset. (D) This figure shows molecular function enrichment analysis of downregulated DEGs in integrated dataset. (E) This figure shows cellular component enrichment analysis of upregulated DEGs in the GSE40435 dataset. (F) This figure shows cellular component enrichment analysis of downregulated DEGs in the GSE40435 dataset. (G) This figure shows molecular function enrichment analysis of upregulated DEGs in the GSE40435 dataset. (H) This figure shows molecular function enrichment analysis of downregulated DEGs in the GSE40435 dataset. Figure S5 (A) This figure shows KEGG pathway enrichment analysis of upregulated DEGs in integrated dataset. (B) This figure shows KEGG pathway enrichment analysis of downregulated DEGs in integrated dataset. (C) This figure shows KEGG pathway enrichment analysis of upregulated DEGs in the GSE40435 dataset. (D) This figure shows KEGG pathway enrichment analysis of downregulated DEGs in the GSE40435 dataset. [file IJCB-2026-5567255-s003.zip › Supplementary Figures Caption.docx]

| 1. 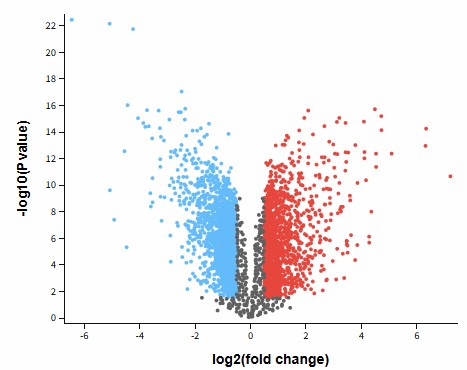 | 1. 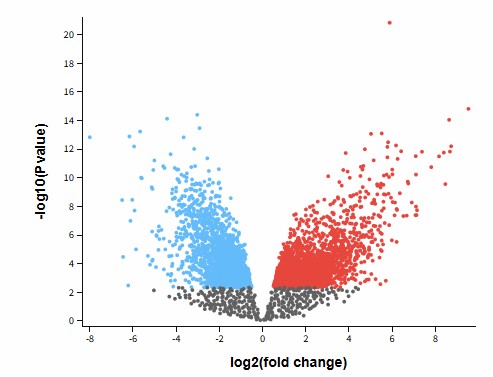 |
| --- | --- |
| 1. 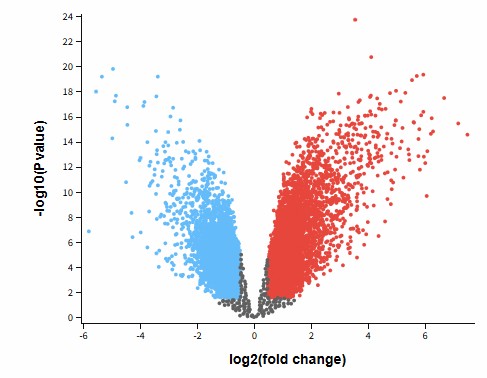 | 1. 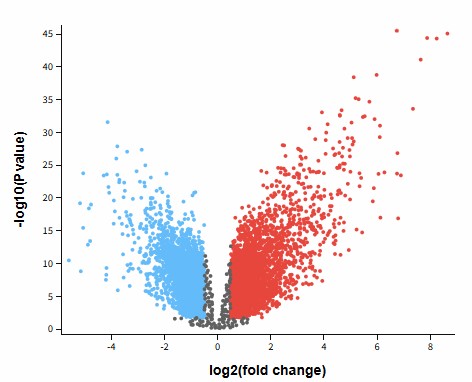 |
| 1. 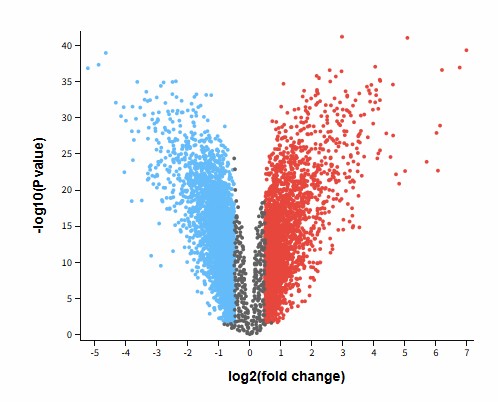 | 1. 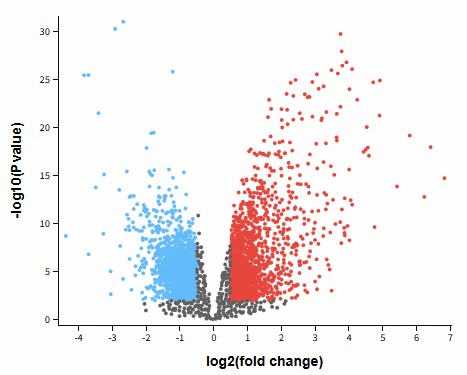 |
| 1. 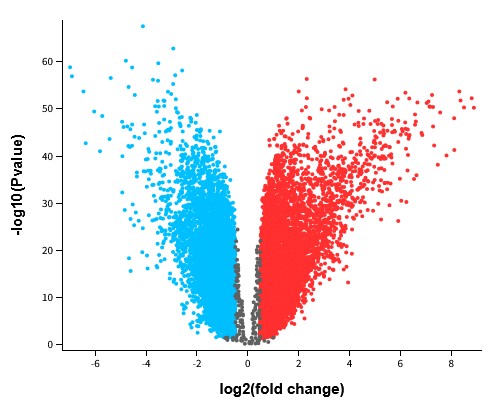 | 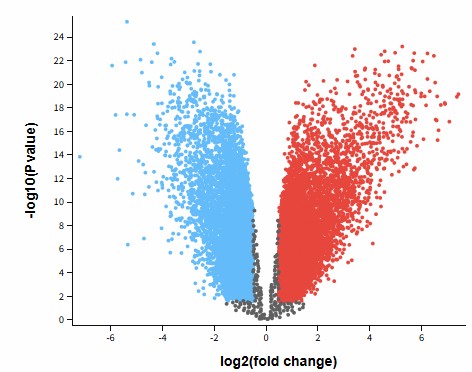 |
| 1. 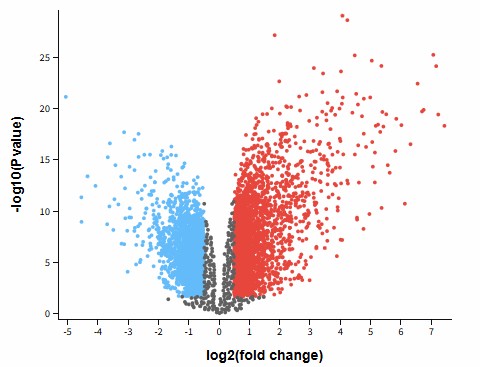 | 1. 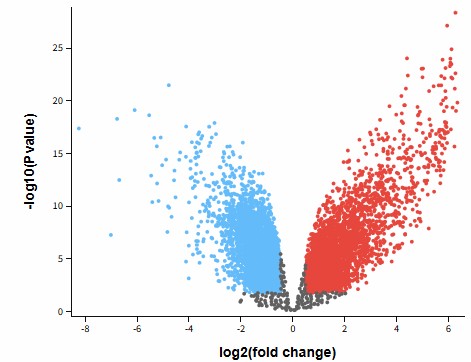 |
| 1. 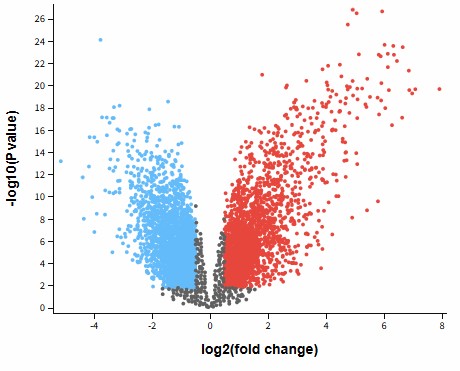 | 1. 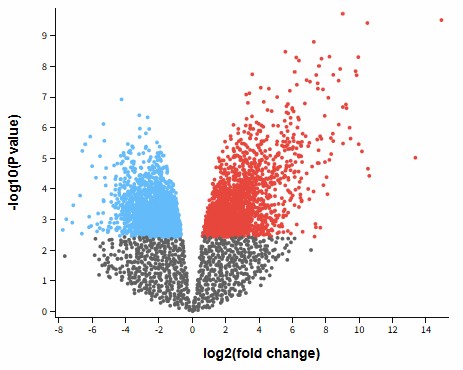 |

**Supplementary Figure S1:** Differential gene expression (DEG) analysis results for each dataset, performed independently using the GEO2R online tool. Panels A-L display genes with an adjusted p-value < 0.05 and |log₂ fold change| > 0.5, indicating significant differential expression across the conditions analyzed. The datasets corresponding to each panel are A) GSE11024, B) GSE11151, C) GSE16441, D) GSE36895, E) GSE46699, F) GSE53000, G) GSE53757, H) GSE66272, I) GSE68417, J) GSE71963, K) GSE76351, L) GSE168845.

| A) 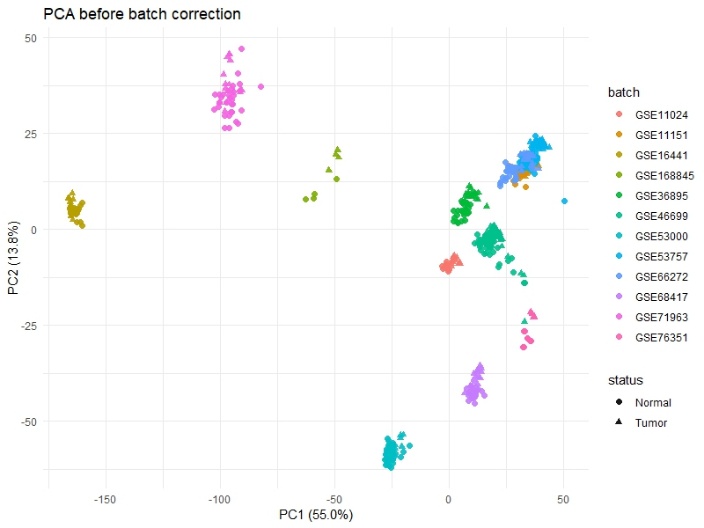 | 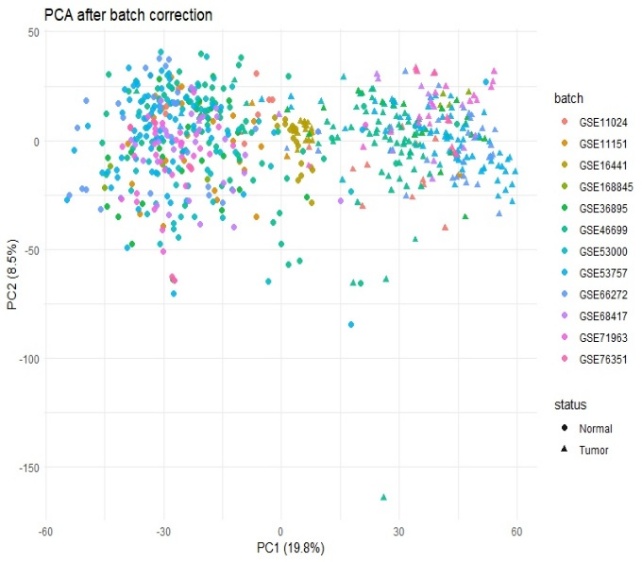B) |
| --- | --- |

**Supplementary Figure S2.** Principal Component Analysis (PCA) before and after batch-effect correction. A) PCA plot showing sample separation based on dataset origin, indicating the presence of batch effects. B) PCA plot after batch-effect correction, showing improved blending of datasets and moderate separation between tumor and normal samples.


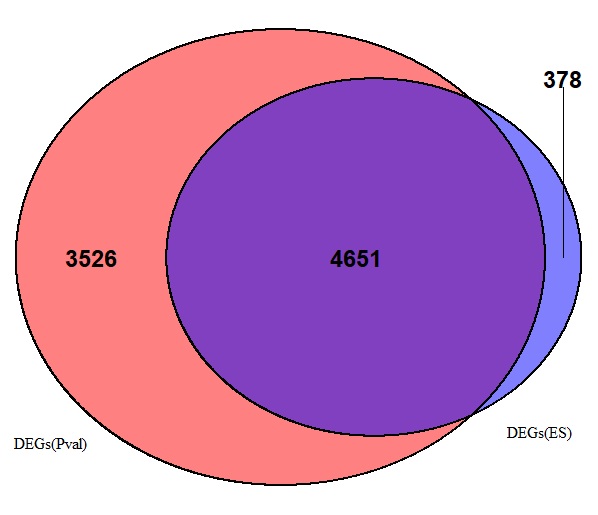


**Supplementary Figure S3.** Venn diagram showing the overlap of differentially expressed genes (DEGs) identified by combining p-values using Fisher’s sum of logs method and by combining individual effect sizes using a random effects model.

| A) 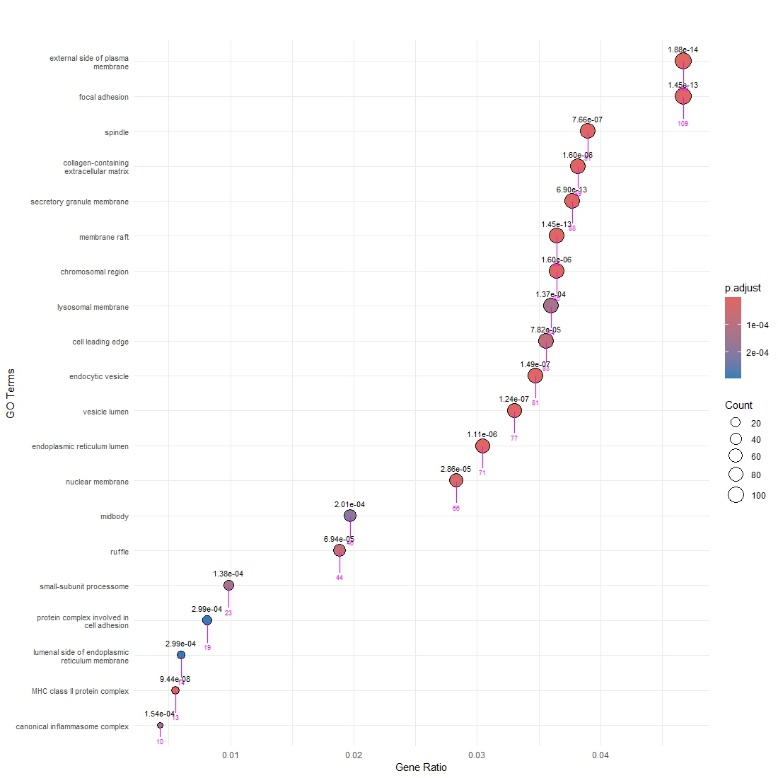 | B) 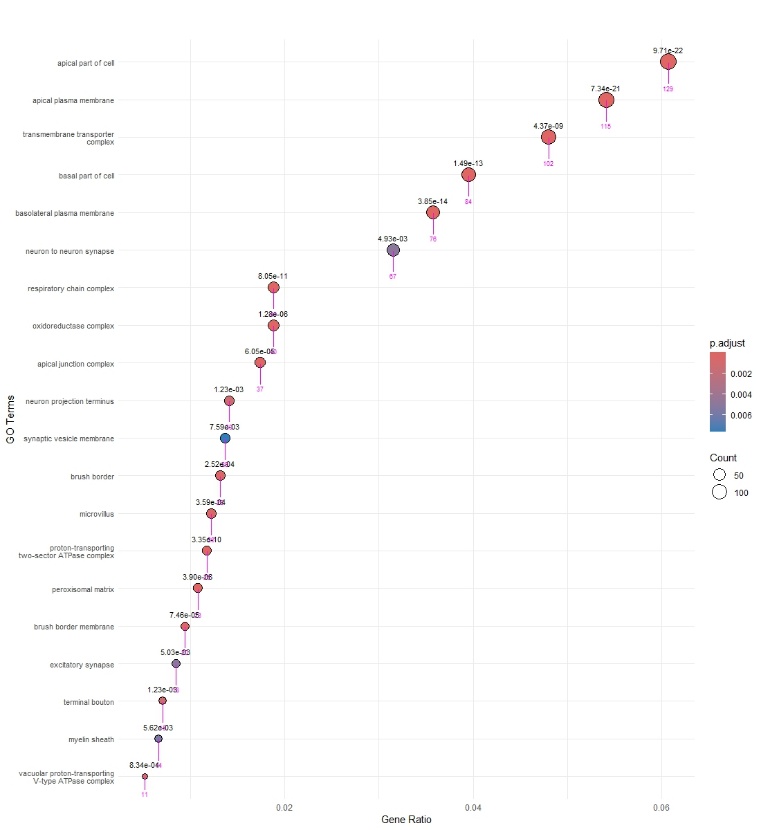 |
| --- | --- |
| C)  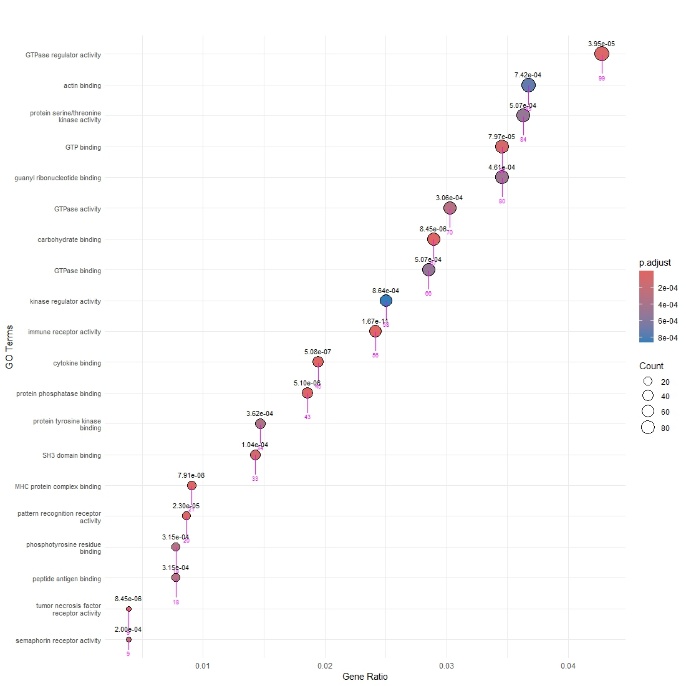 | D)  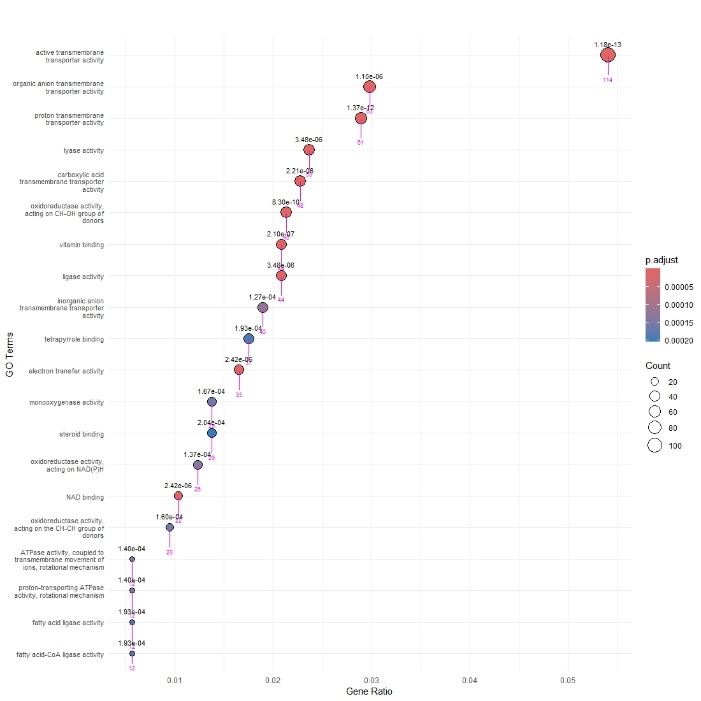 |
| 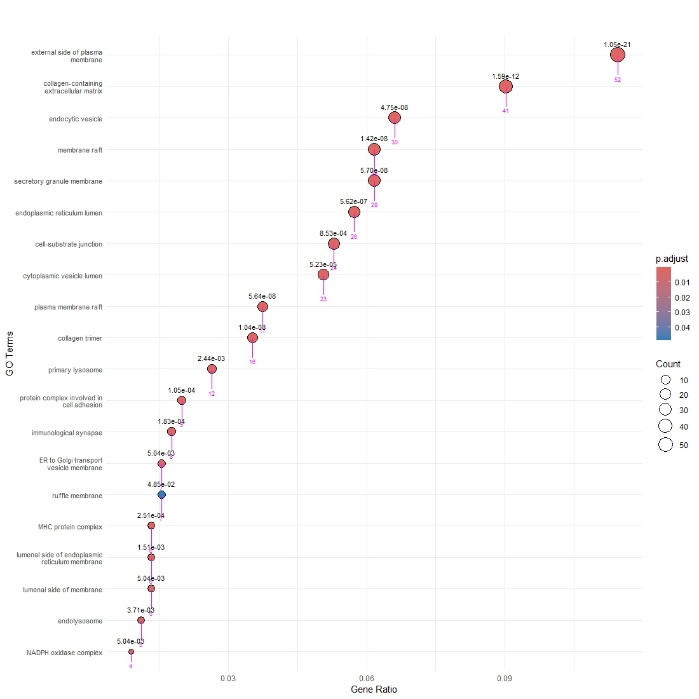E) | F) 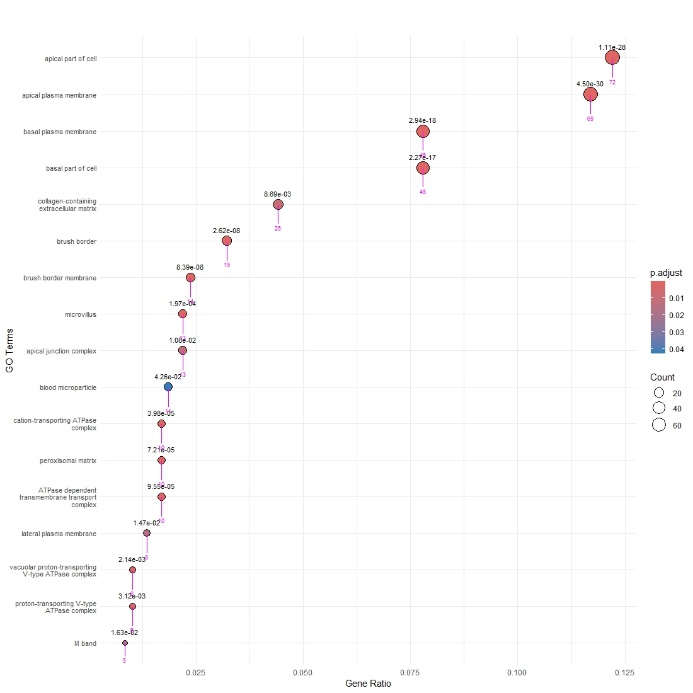 |
| 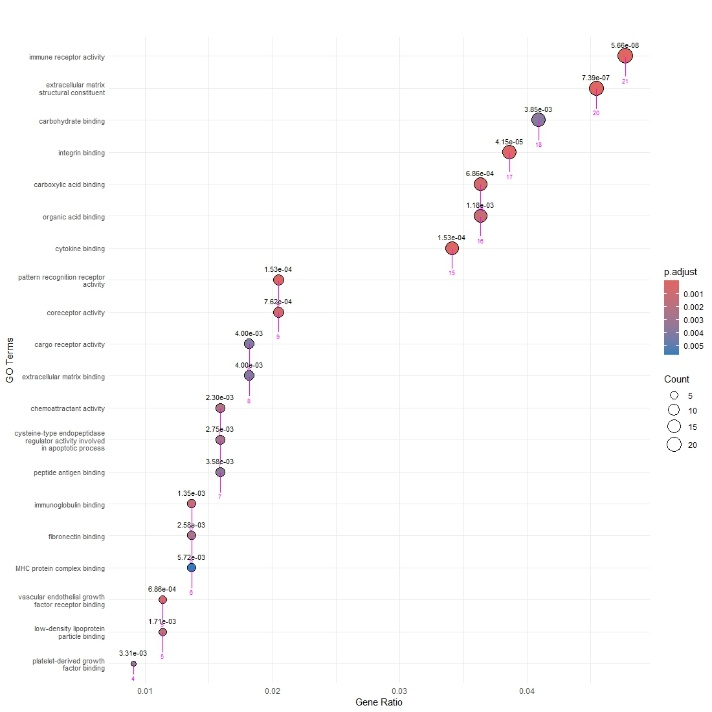G) | 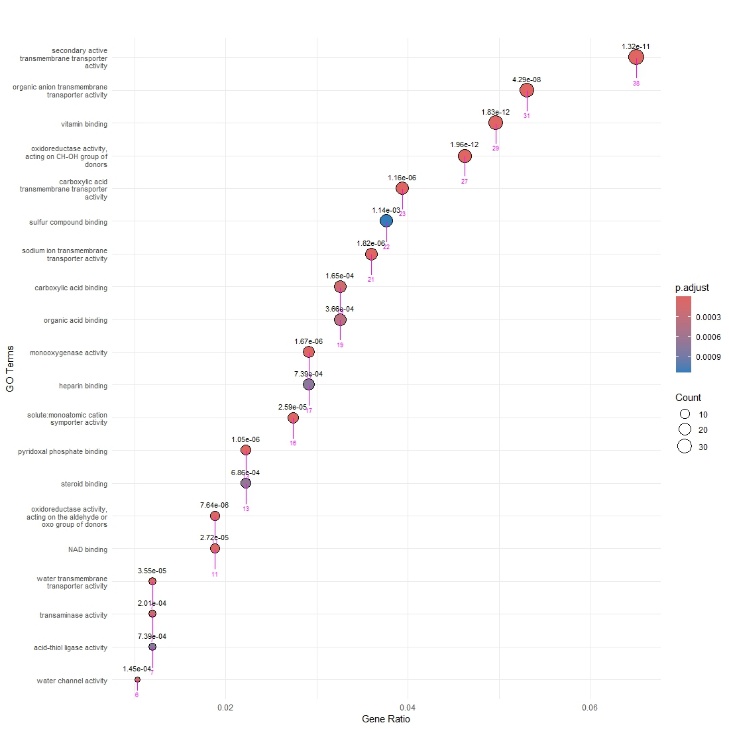H) |

**Supplementary Figure S4**. GO-CC and GO-MF enrichment analysis of DEGs from integrated (A-B) and validation datasets (E-H).
(A) GO-CC analysis of upregulated DEGs reveals enrichment in pathways related to cell adhesion, migration, and cytoskeleton remodeling.
(B) GO-CC analysis of downregulated DEGs shows enrichment in pathways related to cell polarity and membrane organization.
(C) GO-MF analysis of upregulated DEGs indicates enrichment in GTPase activity, actin binding, and receptor-cytokine interactions.
(D) GO-MF analysis of downregulated DEGs highlights enrichment in transmembrane transporter activities and metabolic energy production pathways.
(E) GO-CC analysis of upregulated DEGs reveals enrichment in involved in cell adhesion, extracellular matrix organization, vesicular transport, and cytoskeleton remodeling.
(F) GO-CC analysis of downregulated DEGs reveals that downregulated DEGs were most significantly associated with pathways involved in cell polarity, membrane organization, and specialized membrane structures.
(G) GO-MF analysis of upregulated DEGs indicates enrichment in GTPase activity, GTP binding, receptor-cytokine interactions, and extracellular matrix binding activities.

(H) GO-MF analysis of downregulated DEGs highlights enrichment in transmembrane transporter activities, including those for organic anions, carboxylic acids, and ions, as well as metabolic functions such as oxidoreductase, lyase, and lipid transporter activities.
The top 20 enriched GO terms, ranked by gene count, are presented in each plot. The size of each dot corresponds to the number of genes associated with the respective GO term (pink arrows), and the color reflects the adjusted p-value. The numbers above each dot represent the actual adjusted p-value. The x-axis represents the gene proportion within each GO term relative to the total number of genes in that term, and the y-axis lists the enriched GO terms from the CC and MF categories. A complete list of GO terms for integrated dataset can be found in Supplementary Tables 7-10. A complete list of GO terms for validation dataset can be found in Supplementary Tables 11-14. GO, Gene Ontology; DEGs, differentially expressed genes; CC, cellular component; MF, molecular function.

| A) 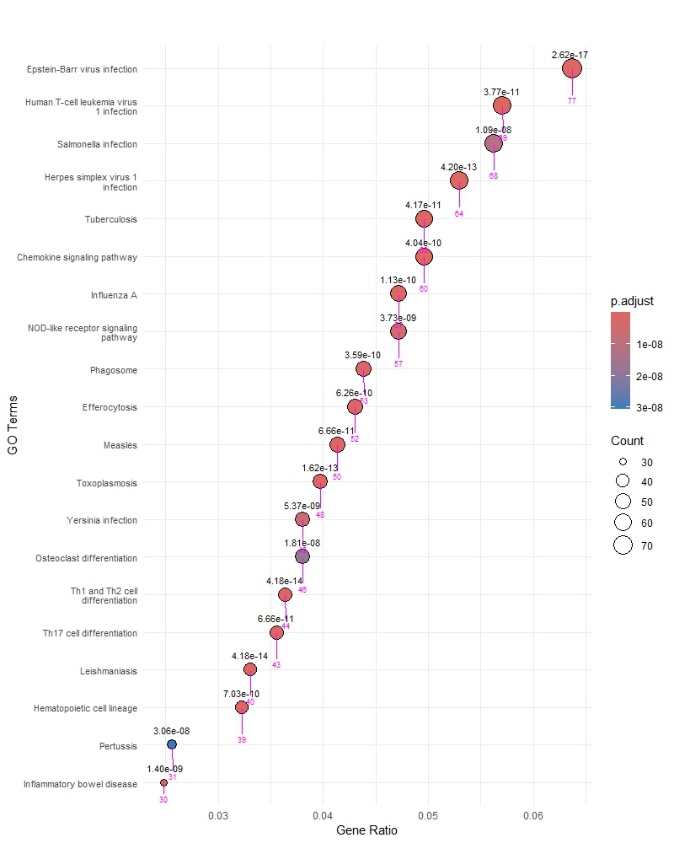 | 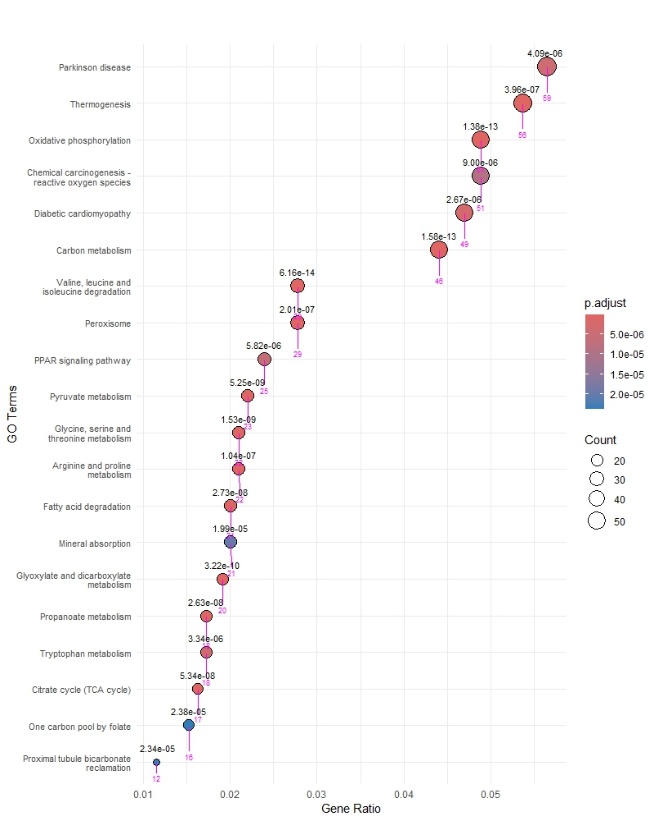B) |
| --- | --- |
| C)  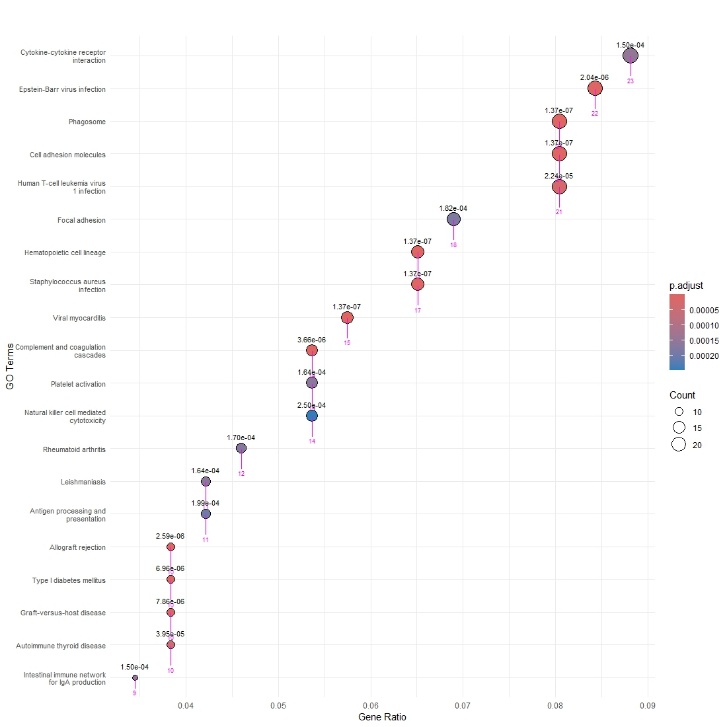 | 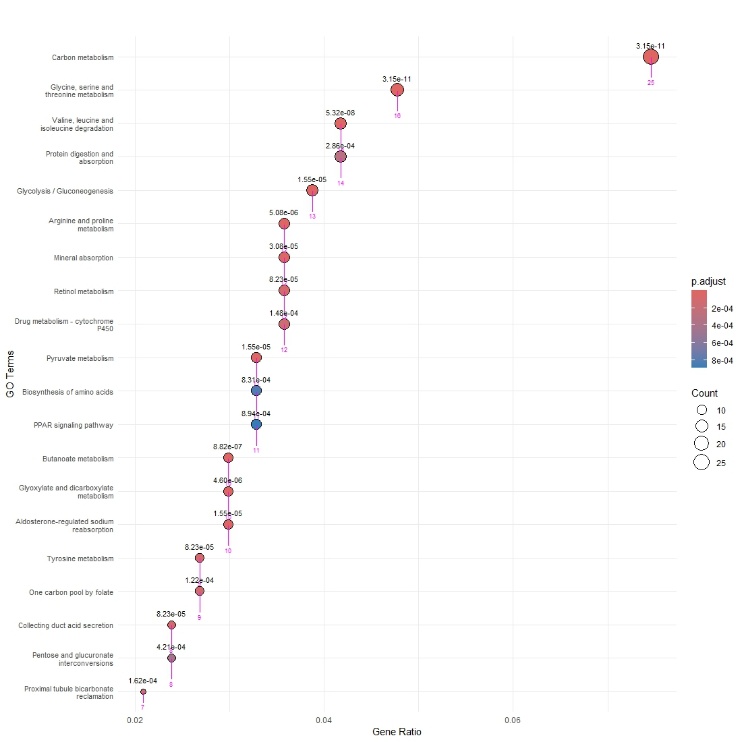D) |

**Supplementary Figure S5**. KEGG pathway enrichment analysis of DEGs from integrated (A, B) and validation datasets (C, D).
(A) Upregulated DEGs in the integrated dataset are enriched in immune system and microbial defense pathways.
(B) Downregulated DEGs in the integrated dataset are primarily associated with amino acid metabolism.
(C) Upregulated DEGs in the validation dataset are primarily associated with immune system and microbial defense pathways.
(D) Downregulated DEGs in the validation dataset show amino acid metabolism as a significantly enriched pathway.

The top 20 enriched KEGGs, ranked by gene count, are presented in each plot. The size of each dot corresponds to the number of genes associated with the respective KEGG pathway (pink arrows), and the color reflects the adjusted p-value. The numbers above each dot represent the actual adjusted p-value. The x-axis represents the gene proportion within each KEGG pathway relative to the total number of genes in that term, and the y-axis lists the enriched KEGG pathway from the CC and MF categories. A complete list of KEGG pathways for integrated dataset can be found in Supplementary Tables 15,16. A complete list of KEGG pathways for validation dataset can be found in Supplementary Tables 17, 18. KEGG, Kyoto Encyclopedia of Genes and Genomes; DEGs, differentially expressed genes.


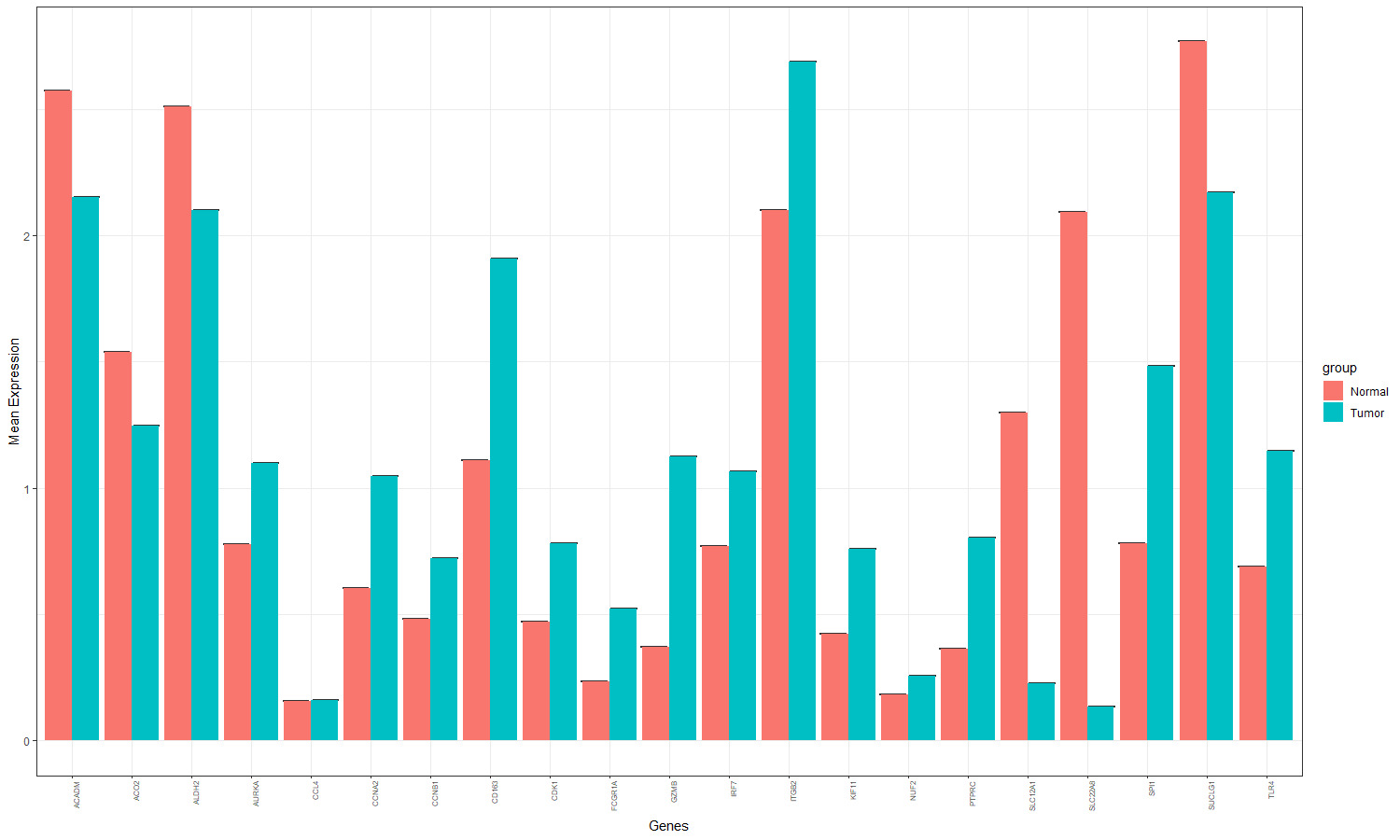


**Supplementary Figure S6.** Illustrates the expression profiles of 21 pivotal genes within the GSE40435 validation dataset.
