# Supplementary figures and images for: Integrative Meta‐Analysis and WGCNA Reveal Candidate Diagnostic Hub Genes in Clear Cell Carcinoma
Source: Int J Cell Biol. 2026 Feb 2;2026:5567255. doi: 10.1155/ijcb/5567255 (PMC12865128; doi:10.1155/ijcb/5567255)

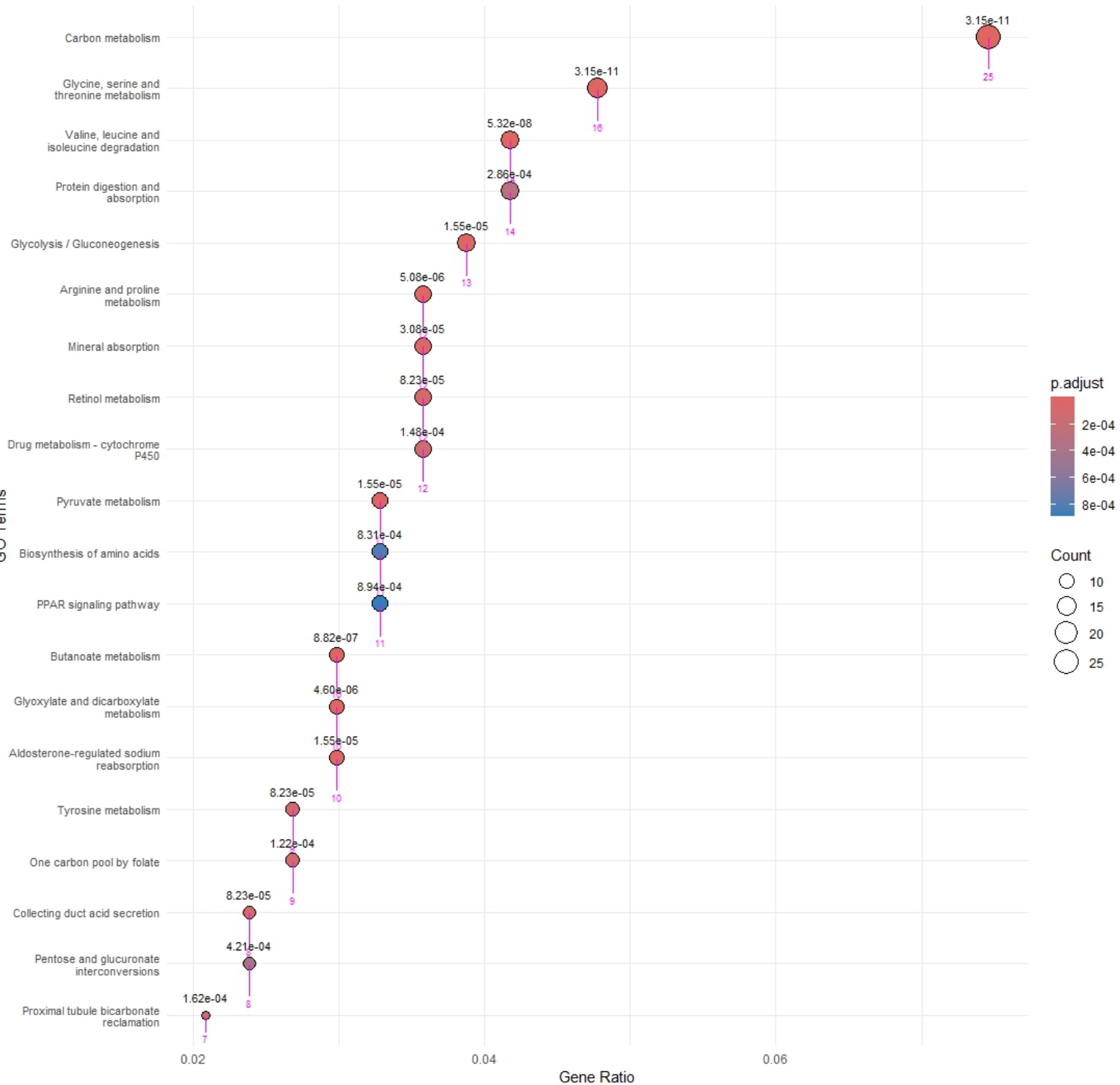

Supplement: Supplementary file 1 — Supporting Information 1 Figure S1. Volcano plots depicting differential gene expression (DEG) analysis results for each dataset, performed independently using the GEO2R online tool. Figure S2. Principal component analysis (PCA) before and after batch‐effect correction. Figure S3. Venn diagram showing the overlap of differentially expressed genes (DEGs) identified by combining p values using Fisher′s sum of logs method and by combining individual effect sizes using a random effects model. Figure S4. (A) This figure shows cellular component enrichment analysis of upregulated DEGs in integrated dataset. (B) This figure shows cellular component enrichment analysis of downregulated DEGs in integrated dataset. (C) This figure shows molecular function enrichment analysis of upregulated DEGs in integrated dataset. (D) This figure shows molecular function enrichment analysis of downregulated DEGs in integrated dataset. (E) This figure shows cellular component enrichment analysis of upregulated DEGs in the GSE40435 dataset. (F) This figure shows cellular component enrichment analysis of downregulated DEGs in the GSE40435 dataset. (G) This figure shows molecular function enrichment analysis of upregulated DEGs in the GSE40435 dataset. (H) This figure shows molecular function enrichment analysis of downregulated DEGs in the GSE40435 dataset. Figure S5 (A) This figure shows KEGG pathway enrichment analysis of upregulated DEGs in integrated dataset. (B) This figure shows KEGG pathway enrichment analysis of downregulated DEGs in integrated dataset. (C) This figure shows KEGG pathway enrichment analysis of upregulated DEGs in the GSE40435 dataset. (D) This figure shows KEGG pathway enrichment analysis of downregulated DEGs in the GSE40435 dataset. [file IJCB-2026-5567255-s003.zip › Supplementary Figure S5 D.pdf]

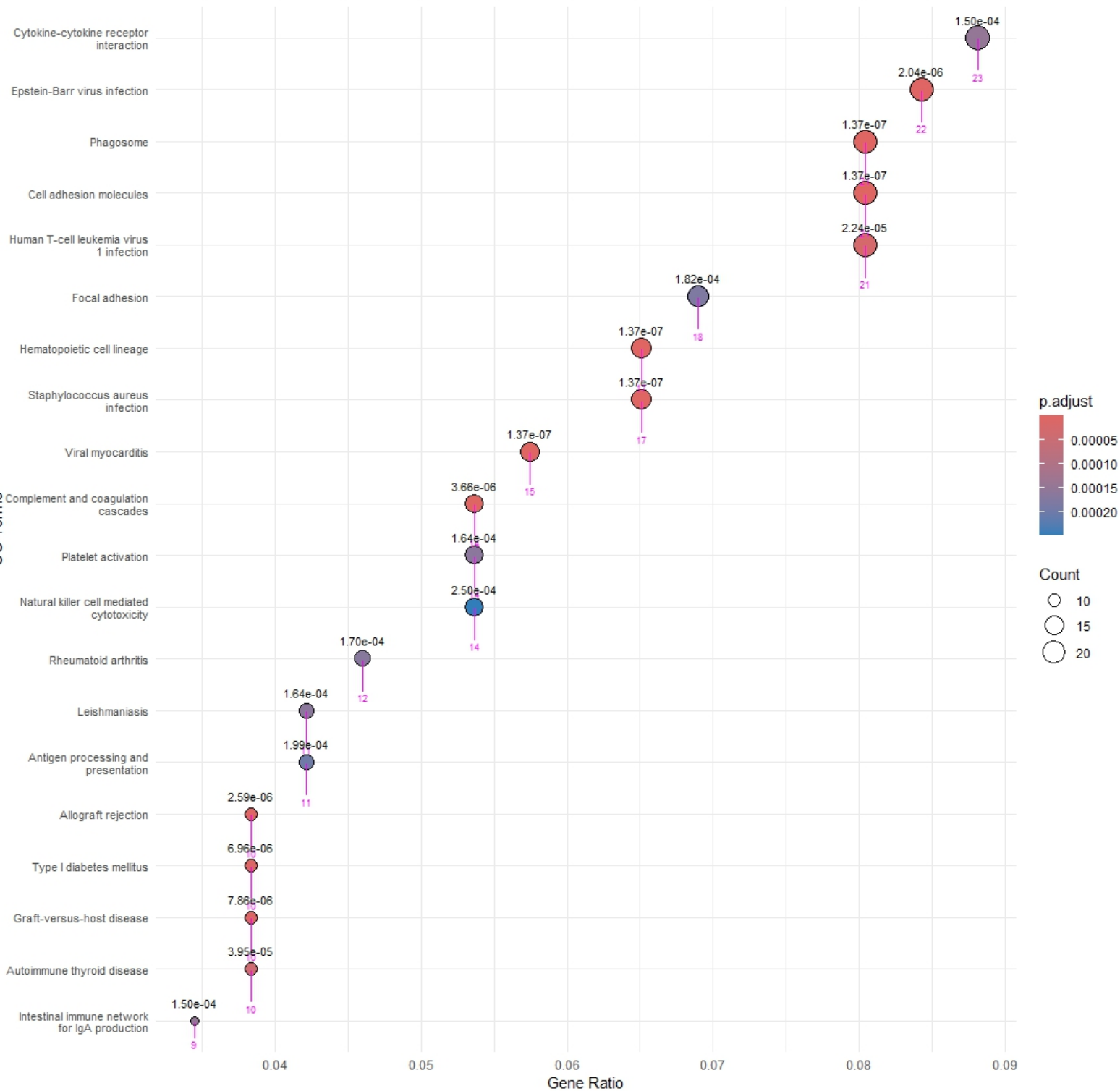

Supplement: Supplementary file 1 — Supporting Information 1 Figure S1. Volcano plots depicting differential gene expression (DEG) analysis results for each dataset, performed independently using the GEO2R online tool. Figure S2. Principal component analysis (PCA) before and after batch‐effect correction. Figure S3. Venn diagram showing the overlap of differentially expressed genes (DEGs) identified by combining p values using Fisher′s sum of logs method and by combining individual effect sizes using a random effects model. Figure S4. (A) This figure shows cellular component enrichment analysis of upregulated DEGs in integrated dataset. (B) This figure shows cellular component enrichment analysis of downregulated DEGs in integrated dataset. (C) This figure shows molecular function enrichment analysis of upregulated DEGs in integrated dataset. (D) This figure shows molecular function enrichment analysis of downregulated DEGs in integrated dataset. (E) This figure shows cellular component enrichment analysis of upregulated DEGs in the GSE40435 dataset. (F) This figure shows cellular component enrichment analysis of downregulated DEGs in the GSE40435 dataset. (G) This figure shows molecular function enrichment analysis of upregulated DEGs in the GSE40435 dataset. (H) This figure shows molecular function enrichment analysis of downregulated DEGs in the GSE40435 dataset. Figure S5 (A) This figure shows KEGG pathway enrichment analysis of upregulated DEGs in integrated dataset. (B) This figure shows KEGG pathway enrichment analysis of downregulated DEGs in integrated dataset. (C) This figure shows KEGG pathway enrichment analysis of upregulated DEGs in the GSE40435 dataset. (D) This figure shows KEGG pathway enrichment analysis of downregulated DEGs in the GSE40435 dataset. [file IJCB-2026-5567255-s003.zip › Supplementary Figure S5 C.pdf]

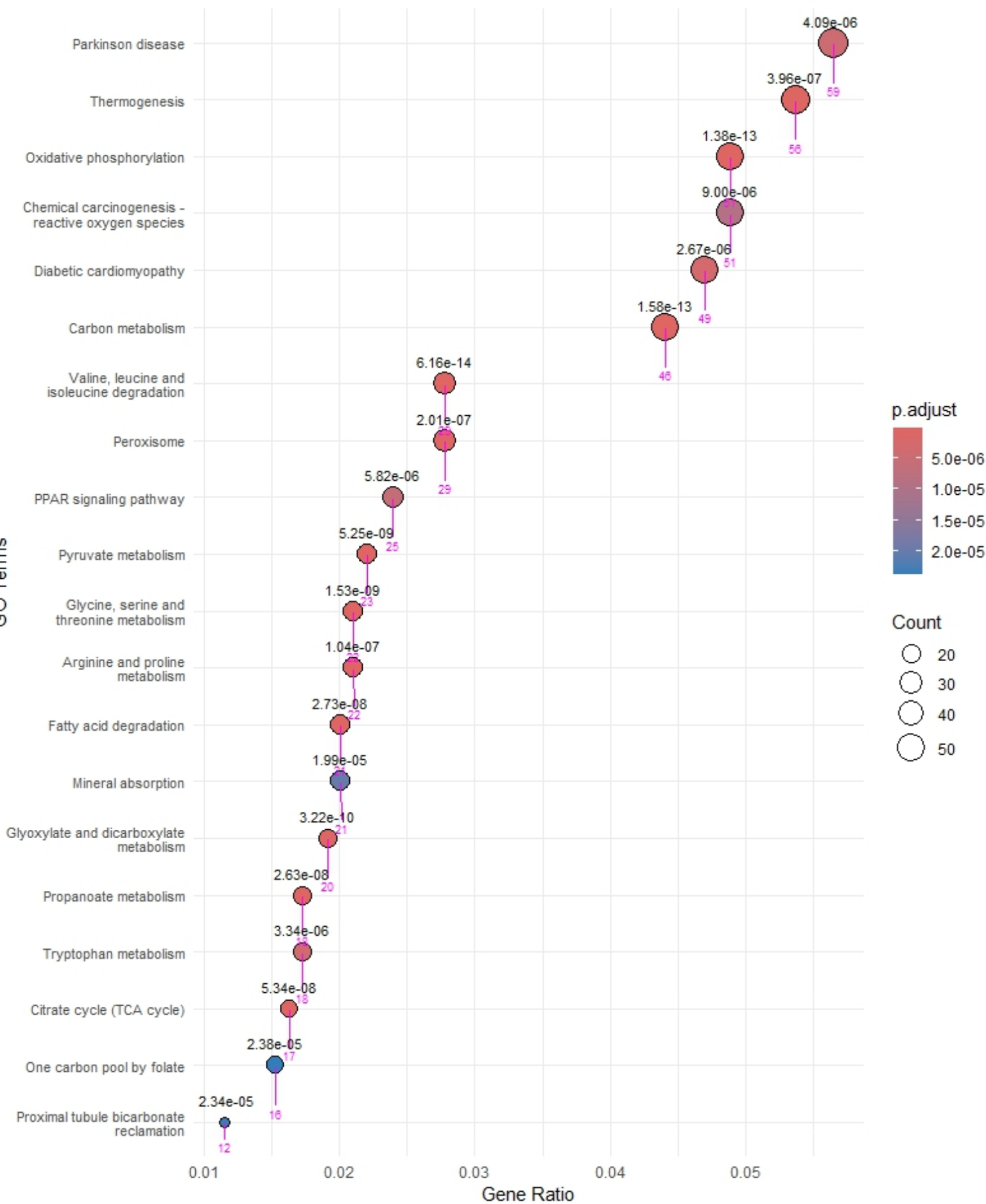

Supplement: Supplementary file 1 — Supporting Information 1 Figure S1. Volcano plots depicting differential gene expression (DEG) analysis results for each dataset, performed independently using the GEO2R online tool. Figure S2. Principal component analysis (PCA) before and after batch‐effect correction. Figure S3. Venn diagram showing the overlap of differentially expressed genes (DEGs) identified by combining p values using Fisher′s sum of logs method and by combining individual effect sizes using a random effects model. Figure S4. (A) This figure shows cellular component enrichment analysis of upregulated DEGs in integrated dataset. (B) This figure shows cellular component enrichment analysis of downregulated DEGs in integrated dataset. (C) This figure shows molecular function enrichment analysis of upregulated DEGs in integrated dataset. (D) This figure shows molecular function enrichment analysis of downregulated DEGs in integrated dataset. (E) This figure shows cellular component enrichment analysis of upregulated DEGs in the GSE40435 dataset. (F) This figure shows cellular component enrichment analysis of downregulated DEGs in the GSE40435 dataset. (G) This figure shows molecular function enrichment analysis of upregulated DEGs in the GSE40435 dataset. (H) This figure shows molecular function enrichment analysis of downregulated DEGs in the GSE40435 dataset. Figure S5 (A) This figure shows KEGG pathway enrichment analysis of upregulated DEGs in integrated dataset. (B) This figure shows KEGG pathway enrichment analysis of downregulated DEGs in integrated dataset. (C) This figure shows KEGG pathway enrichment analysis of upregulated DEGs in the GSE40435 dataset. (D) This figure shows KEGG pathway enrichment analysis of downregulated DEGs in the GSE40435 dataset. [file IJCB-2026-5567255-s003.zip › Supplementary Figure S5 B.pdf]

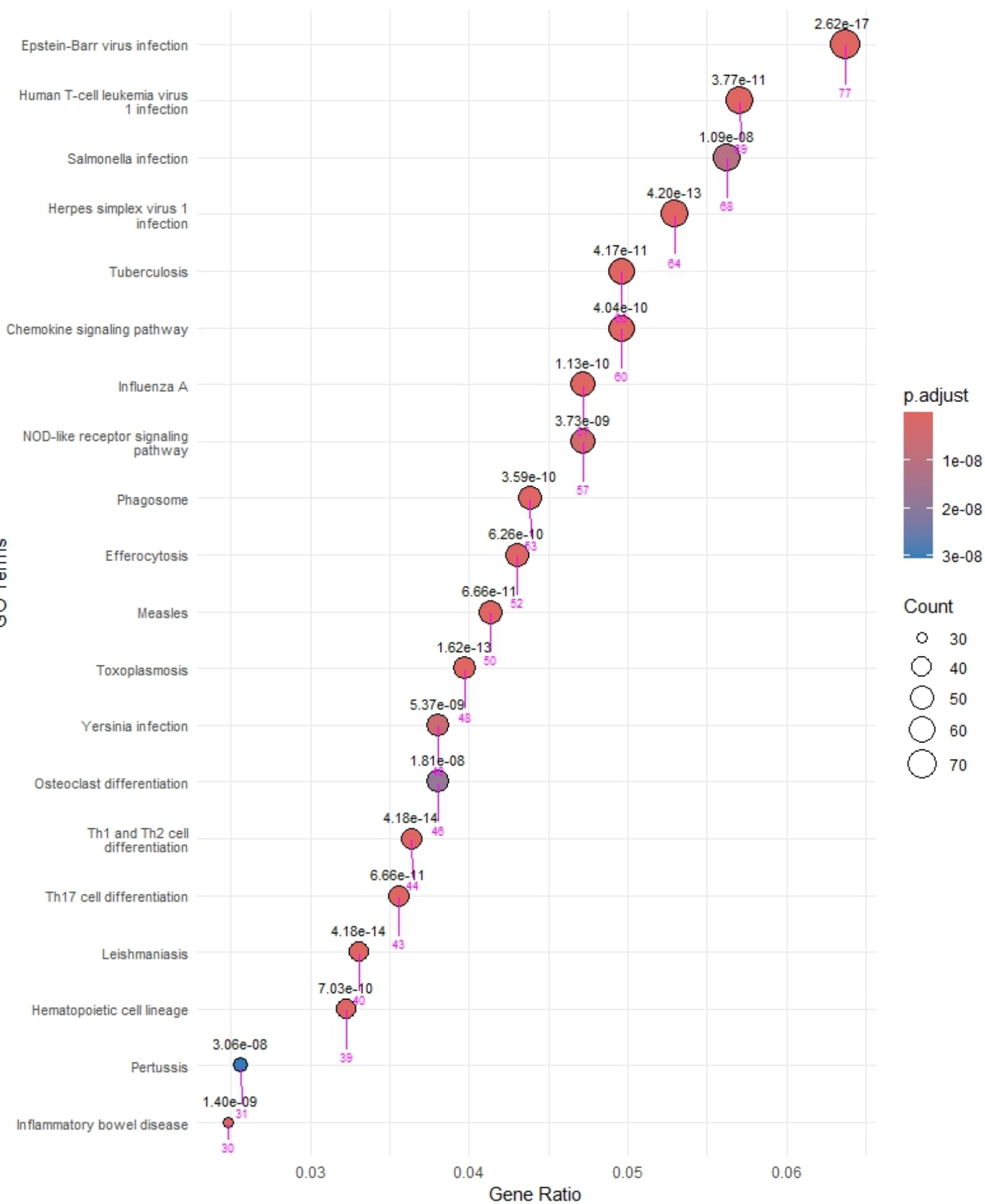

Supplement: Supplementary file 1 — Supporting Information 1 Figure S1. Volcano plots depicting differential gene expression (DEG) analysis results for each dataset, performed independently using the GEO2R online tool. Figure S2. Principal component analysis (PCA) before and after batch‐effect correction. Figure S3. Venn diagram showing the overlap of differentially expressed genes (DEGs) identified by combining p values using Fisher′s sum of logs method and by combining individual effect sizes using a random effects model. Figure S4. (A) This figure shows cellular component enrichment analysis of upregulated DEGs in integrated dataset. (B) This figure shows cellular component enrichment analysis of downregulated DEGs in integrated dataset. (C) This figure shows molecular function enrichment analysis of upregulated DEGs in integrated dataset. (D) This figure shows molecular function enrichment analysis of downregulated DEGs in integrated dataset. (E) This figure shows cellular component enrichment analysis of upregulated DEGs in the GSE40435 dataset. (F) This figure shows cellular component enrichment analysis of downregulated DEGs in the GSE40435 dataset. (G) This figure shows molecular function enrichment analysis of upregulated DEGs in the GSE40435 dataset. (H) This figure shows molecular function enrichment analysis of downregulated DEGs in the GSE40435 dataset. Figure S5 (A) This figure shows KEGG pathway enrichment analysis of upregulated DEGs in integrated dataset. (B) This figure shows KEGG pathway enrichment analysis of downregulated DEGs in integrated dataset. (C) This figure shows KEGG pathway enrichment analysis of upregulated DEGs in the GSE40435 dataset. (D) This figure shows KEGG pathway enrichment analysis of downregulated DEGs in the GSE40435 dataset. [file IJCB-2026-5567255-s003.zip › Supplementary Figure S5 A.pdf]

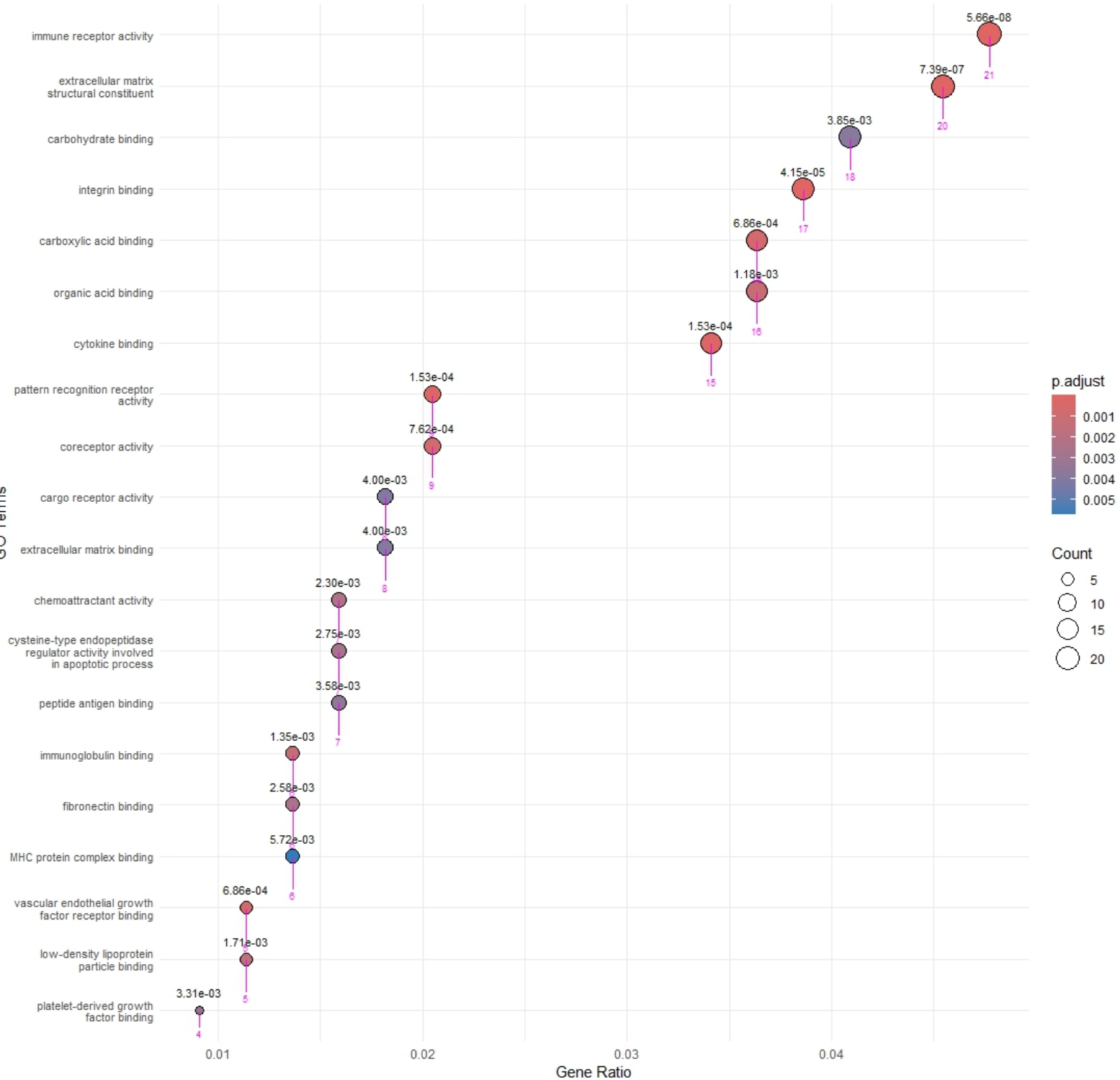

Supplement: Supplementary file 1 — Supporting Information 1 Figure S1. Volcano plots depicting differential gene expression (DEG) analysis results for each dataset, performed independently using the GEO2R online tool. Figure S2. Principal component analysis (PCA) before and after batch‐effect correction. Figure S3. Venn diagram showing the overlap of differentially expressed genes (DEGs) identified by combining p values using Fisher′s sum of logs method and by combining individual effect sizes using a random effects model. Figure S4. (A) This figure shows cellular component enrichment analysis of upregulated DEGs in integrated dataset. (B) This figure shows cellular component enrichment analysis of downregulated DEGs in integrated dataset. (C) This figure shows molecular function enrichment analysis of upregulated DEGs in integrated dataset. (D) This figure shows molecular function enrichment analysis of downregulated DEGs in integrated dataset. (E) This figure shows cellular component enrichment analysis of upregulated DEGs in the GSE40435 dataset. (F) This figure shows cellular component enrichment analysis of downregulated DEGs in the GSE40435 dataset. (G) This figure shows molecular function enrichment analysis of upregulated DEGs in the GSE40435 dataset. (H) This figure shows molecular function enrichment analysis of downregulated DEGs in the GSE40435 dataset. Figure S5 (A) This figure shows KEGG pathway enrichment analysis of upregulated DEGs in integrated dataset. (B) This figure shows KEGG pathway enrichment analysis of downregulated DEGs in integrated dataset. (C) This figure shows KEGG pathway enrichment analysis of upregulated DEGs in the GSE40435 dataset. (D) This figure shows KEGG pathway enrichment analysis of downregulated DEGs in the GSE40435 dataset. [file IJCB-2026-5567255-s003.zip › Supplementary Figure S4 G.pdf]

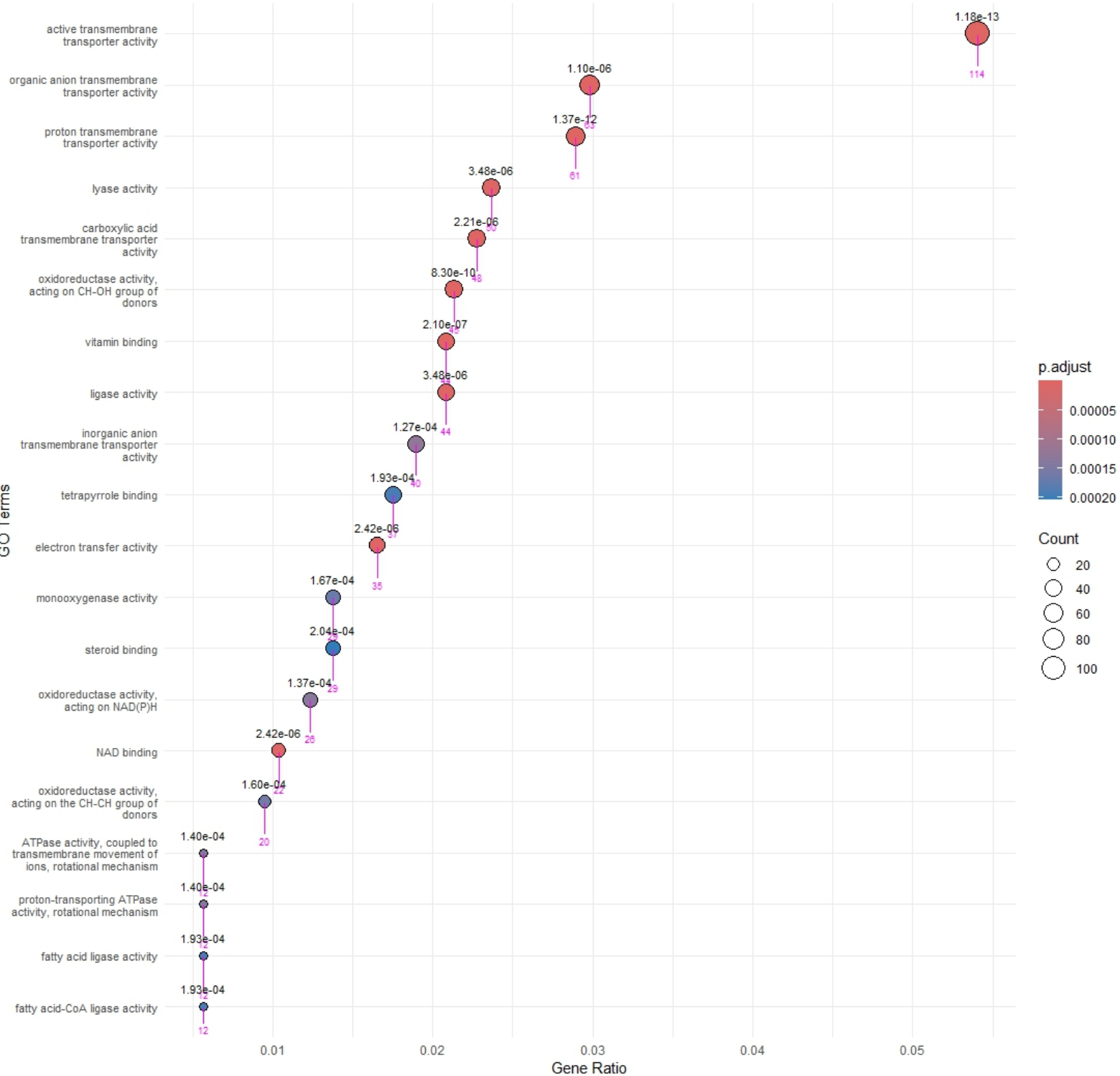

Supplement: Supplementary file 1 — Supporting Information 1 Figure S1. Volcano plots depicting differential gene expression (DEG) analysis results for each dataset, performed independently using the GEO2R online tool. Figure S2. Principal component analysis (PCA) before and after batch‐effect correction. Figure S3. Venn diagram showing the overlap of differentially expressed genes (DEGs) identified by combining p values using Fisher′s sum of logs method and by combining individual effect sizes using a random effects model. Figure S4. (A) This figure shows cellular component enrichment analysis of upregulated DEGs in integrated dataset. (B) This figure shows cellular component enrichment analysis of downregulated DEGs in integrated dataset. (C) This figure shows molecular function enrichment analysis of upregulated DEGs in integrated dataset. (D) This figure shows molecular function enrichment analysis of downregulated DEGs in integrated dataset. (E) This figure shows cellular component enrichment analysis of upregulated DEGs in the GSE40435 dataset. (F) This figure shows cellular component enrichment analysis of downregulated DEGs in the GSE40435 dataset. (G) This figure shows molecular function enrichment analysis of upregulated DEGs in the GSE40435 dataset. (H) This figure shows molecular function enrichment analysis of downregulated DEGs in the GSE40435 dataset. Figure S5 (A) This figure shows KEGG pathway enrichment analysis of upregulated DEGs in integrated dataset. (B) This figure shows KEGG pathway enrichment analysis of downregulated DEGs in integrated dataset. (C) This figure shows KEGG pathway enrichment analysis of upregulated DEGs in the GSE40435 dataset. (D) This figure shows KEGG pathway enrichment analysis of downregulated DEGs in the GSE40435 dataset. [file IJCB-2026-5567255-s003.zip › Supplementary Figure S4 D.pdf]

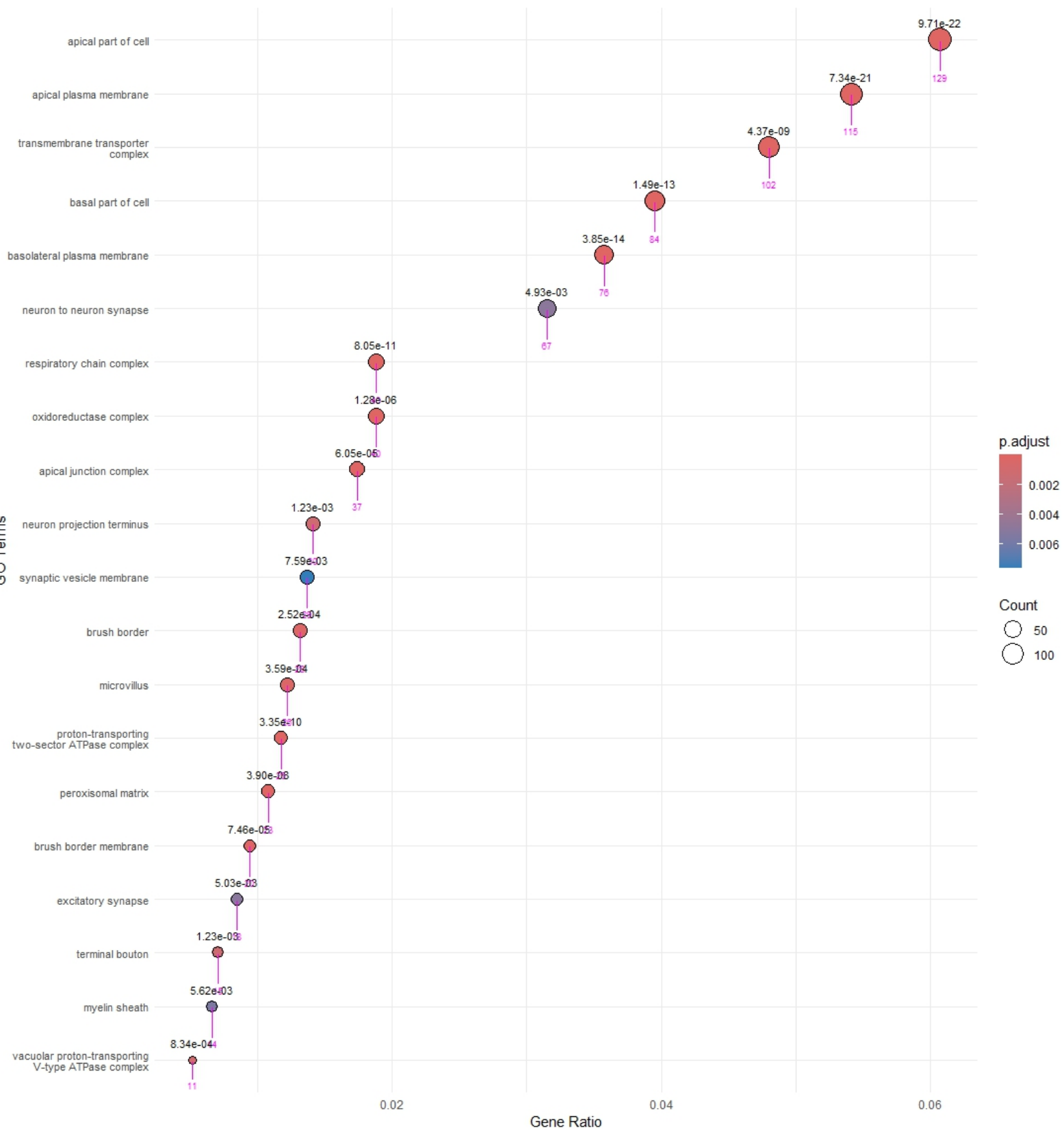

Supplement: Supplementary file 1 — Supporting Information 1 Figure S1. Volcano plots depicting differential gene expression (DEG) analysis results for each dataset, performed independently using the GEO2R online tool. Figure S2. Principal component analysis (PCA) before and after batch‐effect correction. Figure S3. Venn diagram showing the overlap of differentially expressed genes (DEGs) identified by combining p values using Fisher′s sum of logs method and by combining individual effect sizes using a random effects model. Figure S4. (A) This figure shows cellular component enrichment analysis of upregulated DEGs in integrated dataset. (B) This figure shows cellular component enrichment analysis of downregulated DEGs in integrated dataset. (C) This figure shows molecular function enrichment analysis of upregulated DEGs in integrated dataset. (D) This figure shows molecular function enrichment analysis of downregulated DEGs in integrated dataset. (E) This figure shows cellular component enrichment analysis of upregulated DEGs in the GSE40435 dataset. (F) This figure shows cellular component enrichment analysis of downregulated DEGs in the GSE40435 dataset. (G) This figure shows molecular function enrichment analysis of upregulated DEGs in the GSE40435 dataset. (H) This figure shows molecular function enrichment analysis of downregulated DEGs in the GSE40435 dataset. Figure S5 (A) This figure shows KEGG pathway enrichment analysis of upregulated DEGs in integrated dataset. (B) This figure shows KEGG pathway enrichment analysis of downregulated DEGs in integrated dataset. (C) This figure shows KEGG pathway enrichment analysis of upregulated DEGs in the GSE40435 dataset. (D) This figure shows KEGG pathway enrichment analysis of downregulated DEGs in the GSE40435 dataset. [file IJCB-2026-5567255-s003.zip › Supplementary Figure S4 B.pdf]

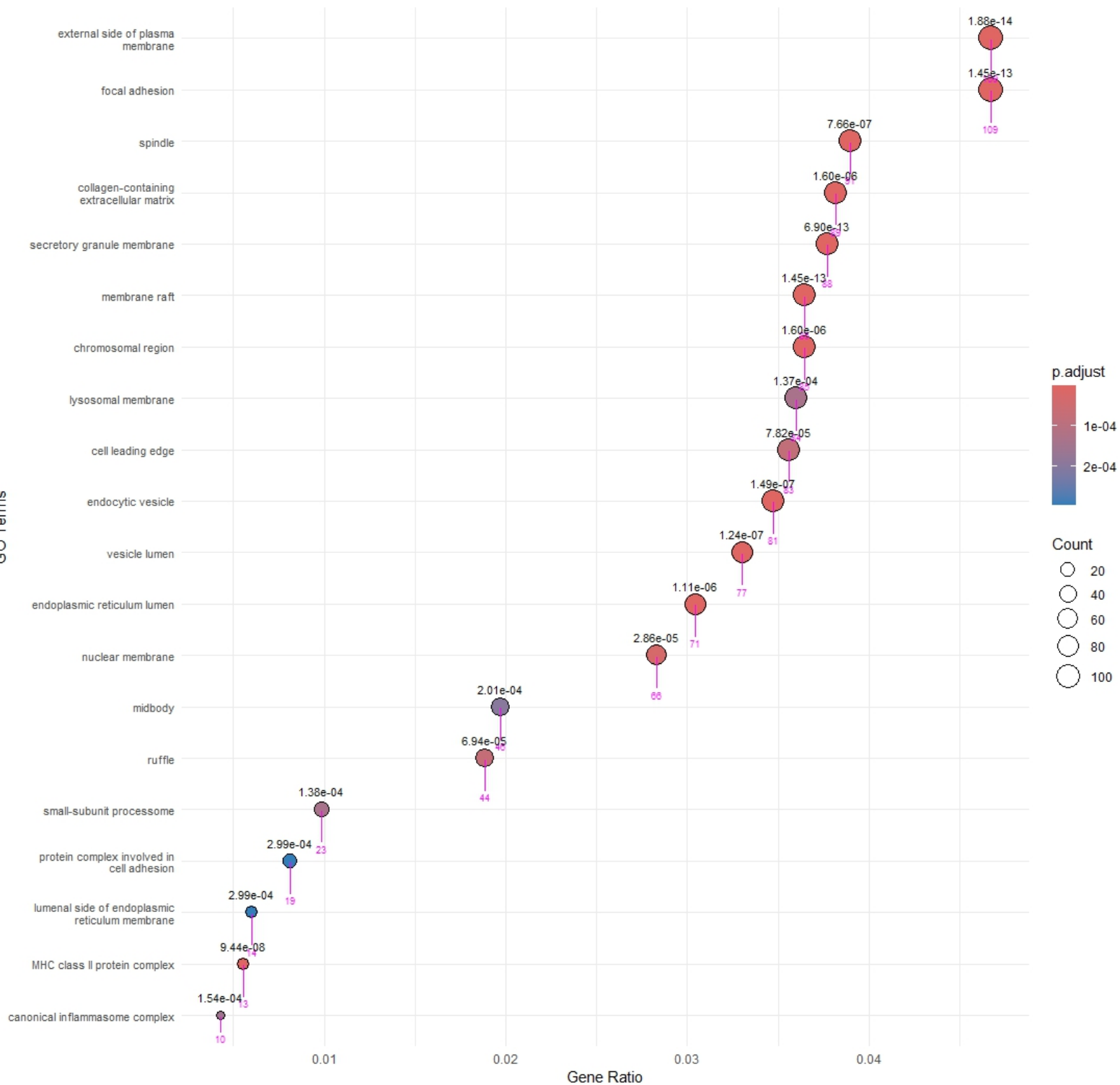

Supplement: Supplementary file 1 — Supporting Information 1 Figure S1. Volcano plots depicting differential gene expression (DEG) analysis results for each dataset, performed independently using the GEO2R online tool. Figure S2. Principal component analysis (PCA) before and after batch‐effect correction. Figure S3. Venn diagram showing the overlap of differentially expressed genes (DEGs) identified by combining p values using Fisher′s sum of logs method and by combining individual effect sizes using a random effects model. Figure S4. (A) This figure shows cellular component enrichment analysis of upregulated DEGs in integrated dataset. (B) This figure shows cellular component enrichment analysis of downregulated DEGs in integrated dataset. (C) This figure shows molecular function enrichment analysis of upregulated DEGs in integrated dataset. (D) This figure shows molecular function enrichment analysis of downregulated DEGs in integrated dataset. (E) This figure shows cellular component enrichment analysis of upregulated DEGs in the GSE40435 dataset. (F) This figure shows cellular component enrichment analysis of downregulated DEGs in the GSE40435 dataset. (G) This figure shows molecular function enrichment analysis of upregulated DEGs in the GSE40435 dataset. (H) This figure shows molecular function enrichment analysis of downregulated DEGs in the GSE40435 dataset. Figure S5 (A) This figure shows KEGG pathway enrichment analysis of upregulated DEGs in integrated dataset. (B) This figure shows KEGG pathway enrichment analysis of downregulated DEGs in integrated dataset. (C) This figure shows KEGG pathway enrichment analysis of upregulated DEGs in the GSE40435 dataset. (D) This figure shows KEGG pathway enrichment analysis of downregulated DEGs in the GSE40435 dataset. [file IJCB-2026-5567255-s003.zip › Supplementary Figure S4 A.pdf]

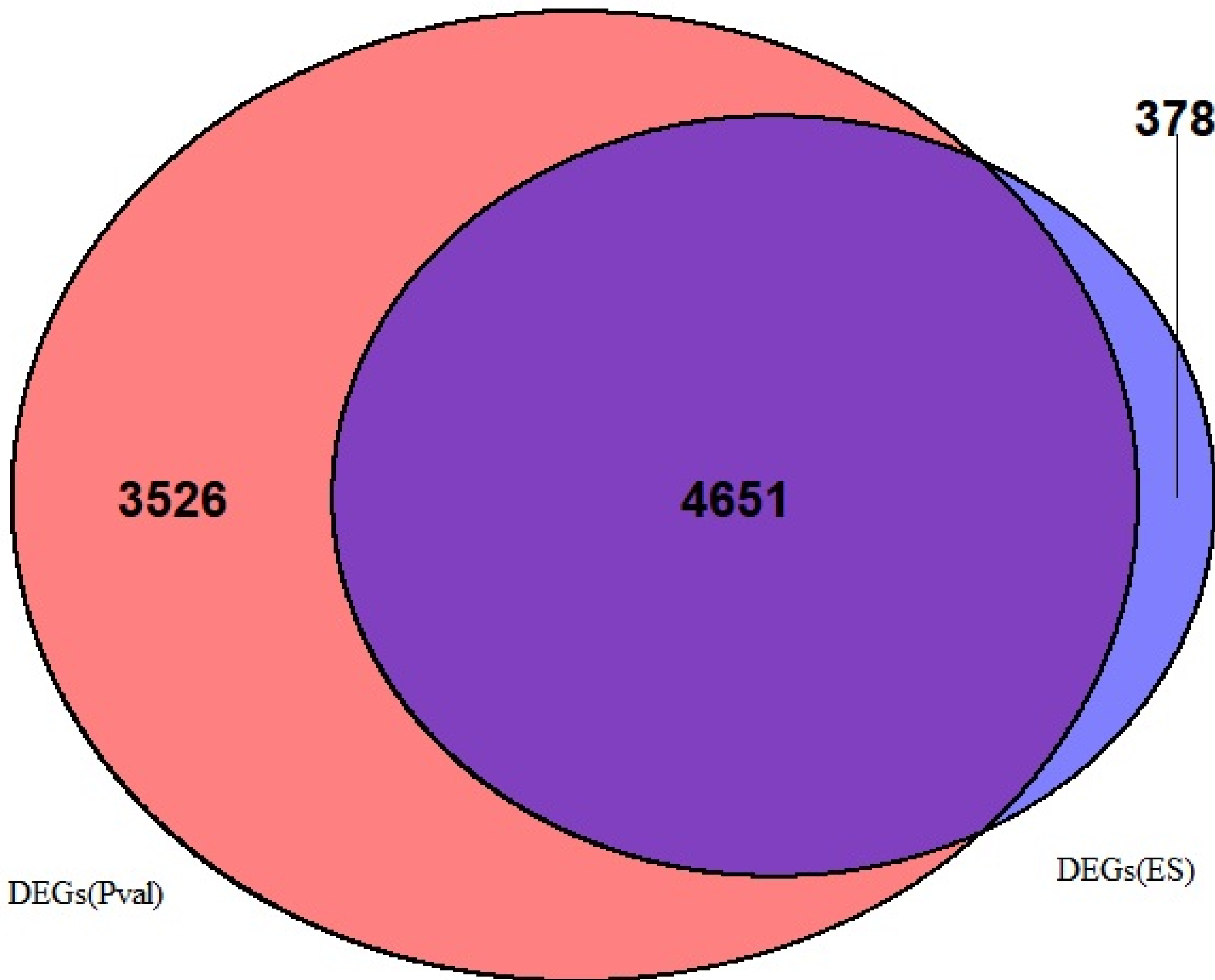

Supplement: Supplementary file 1 — Supporting Information 1 Figure S1. Volcano plots depicting differential gene expression (DEG) analysis results for each dataset, performed independently using the GEO2R online tool. Figure S2. Principal component analysis (PCA) before and after batch‐effect correction. Figure S3. Venn diagram showing the overlap of differentially expressed genes (DEGs) identified by combining p values using Fisher′s sum of logs method and by combining individual effect sizes using a random effects model. Figure S4. (A) This figure shows cellular component enrichment analysis of upregulated DEGs in integrated dataset. (B) This figure shows cellular component enrichment analysis of downregulated DEGs in integrated dataset. (C) This figure shows molecular function enrichment analysis of upregulated DEGs in integrated dataset. (D) This figure shows molecular function enrichment analysis of downregulated DEGs in integrated dataset. (E) This figure shows cellular component enrichment analysis of upregulated DEGs in the GSE40435 dataset. (F) This figure shows cellular component enrichment analysis of downregulated DEGs in the GSE40435 dataset. (G) This figure shows molecular function enrichment analysis of upregulated DEGs in the GSE40435 dataset. (H) This figure shows molecular function enrichment analysis of downregulated DEGs in the GSE40435 dataset. Figure S5 (A) This figure shows KEGG pathway enrichment analysis of upregulated DEGs in integrated dataset. (B) This figure shows KEGG pathway enrichment analysis of downregulated DEGs in integrated dataset. (C) This figure shows KEGG pathway enrichment analysis of upregulated DEGs in the GSE40435 dataset. (D) This figure shows KEGG pathway enrichment analysis of downregulated DEGs in the GSE40435 dataset. [file IJCB-2026-5567255-s003.zip › Supplementary Figure S3.pdf]

PCA after batch correction

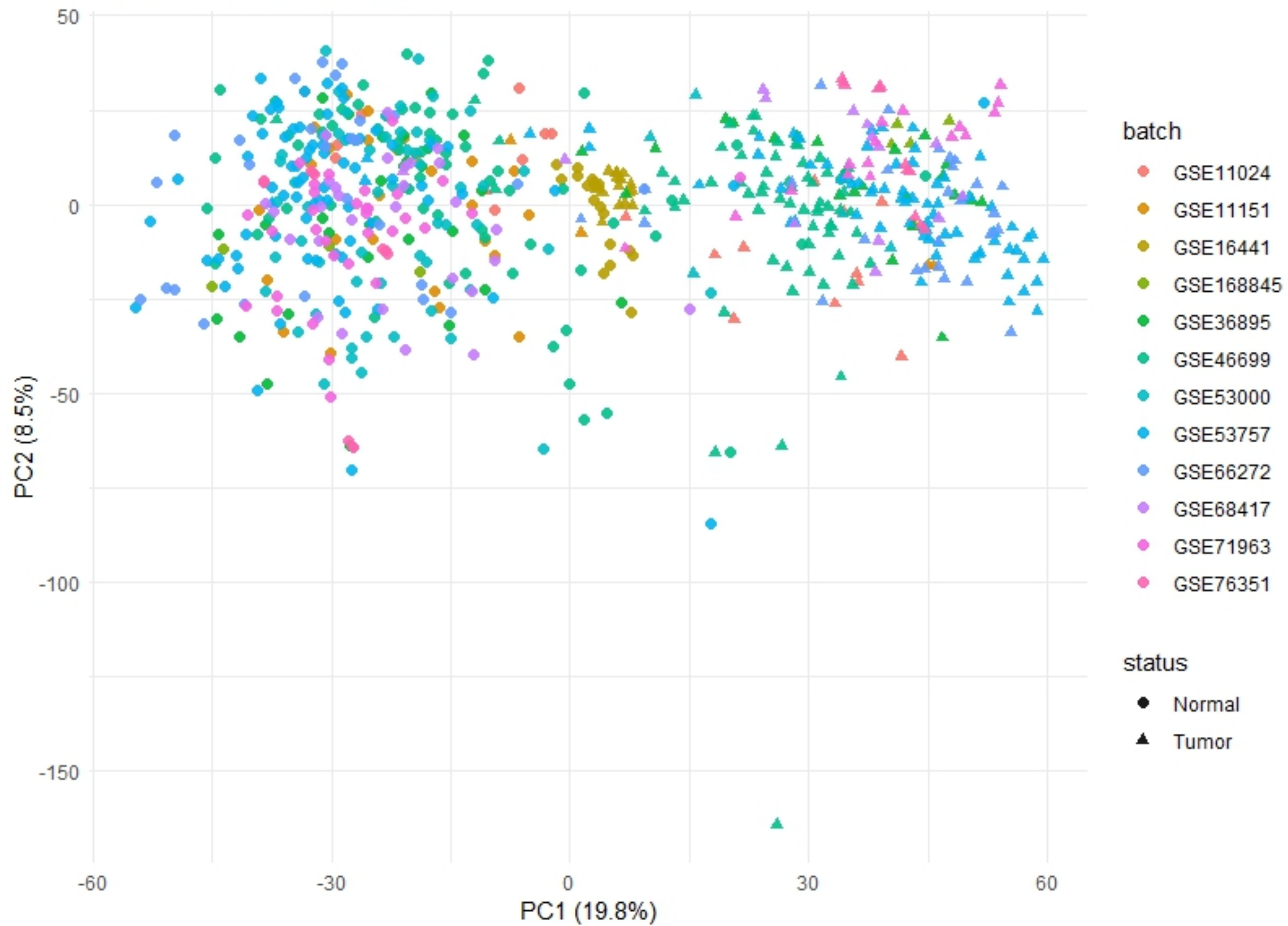

Supplement: Supplementary file 1 — Supporting Information 1 Figure S1. Volcano plots depicting differential gene expression (DEG) analysis results for each dataset, performed independently using the GEO2R online tool. Figure S2. Principal component analysis (PCA) before and after batch‐effect correction. Figure S3. Venn diagram showing the overlap of differentially expressed genes (DEGs) identified by combining p values using Fisher′s sum of logs method and by combining individual effect sizes using a random effects model. Figure S4. (A) This figure shows cellular component enrichment analysis of upregulated DEGs in integrated dataset. (B) This figure shows cellular component enrichment analysis of downregulated DEGs in integrated dataset. (C) This figure shows molecular function enrichment analysis of upregulated DEGs in integrated dataset. (D) This figure shows molecular function enrichment analysis of downregulated DEGs in integrated dataset. (E) This figure shows cellular component enrichment analysis of upregulated DEGs in the GSE40435 dataset. (F) This figure shows cellular component enrichment analysis of downregulated DEGs in the GSE40435 dataset. (G) This figure shows molecular function enrichment analysis of upregulated DEGs in the GSE40435 dataset. (H) This figure shows molecular function enrichment analysis of downregulated DEGs in the GSE40435 dataset. Figure S5 (A) This figure shows KEGG pathway enrichment analysis of upregulated DEGs in integrated dataset. (B) This figure shows KEGG pathway enrichment analysis of downregulated DEGs in integrated dataset. (C) This figure shows KEGG pathway enrichment analysis of upregulated DEGs in the GSE40435 dataset. (D) This figure shows KEGG pathway enrichment analysis of downregulated DEGs in the GSE40435 dataset. [file IJCB-2026-5567255-s003.zip › Supplementary Figure S2 B.pdf]

PCA before batch correction

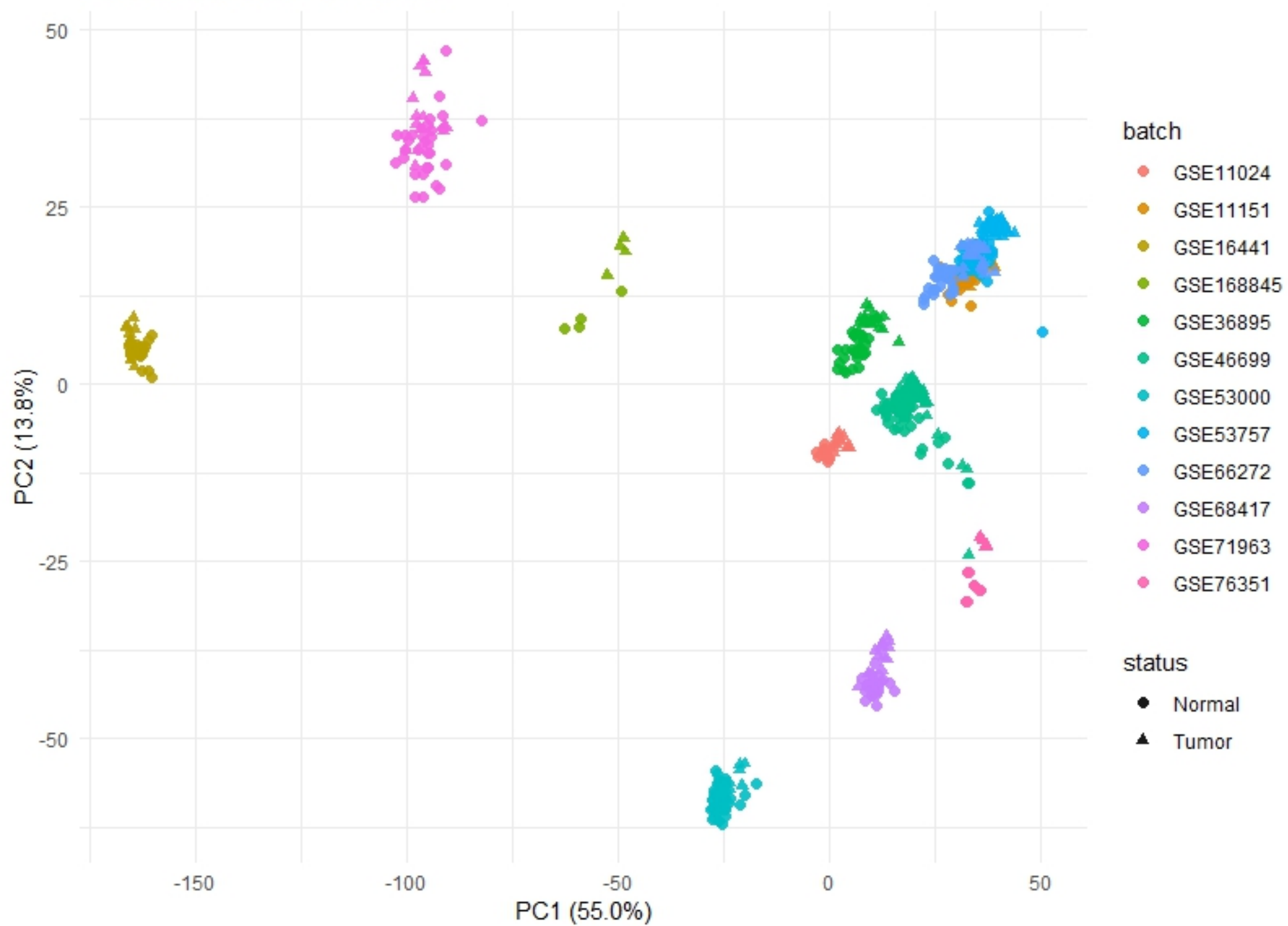

Supplement: Supplementary file 1 — Supporting Information 1 Figure S1. Volcano plots depicting differential gene expression (DEG) analysis results for each dataset, performed independently using the GEO2R online tool. Figure S2. Principal component analysis (PCA) before and after batch‐effect correction. Figure S3. Venn diagram showing the overlap of differentially expressed genes (DEGs) identified by combining p values using Fisher′s sum of logs method and by combining individual effect sizes using a random effects model. Figure S4. (A) This figure shows cellular component enrichment analysis of upregulated DEGs in integrated dataset. (B) This figure shows cellular component enrichment analysis of downregulated DEGs in integrated dataset. (C) This figure shows molecular function enrichment analysis of upregulated DEGs in integrated dataset. (D) This figure shows molecular function enrichment analysis of downregulated DEGs in integrated dataset. (E) This figure shows cellular component enrichment analysis of upregulated DEGs in the GSE40435 dataset. (F) This figure shows cellular component enrichment analysis of downregulated DEGs in the GSE40435 dataset. (G) This figure shows molecular function enrichment analysis of upregulated DEGs in the GSE40435 dataset. (H) This figure shows molecular function enrichment analysis of downregulated DEGs in the GSE40435 dataset. Figure S5 (A) This figure shows KEGG pathway enrichment analysis of upregulated DEGs in integrated dataset. (B) This figure shows KEGG pathway enrichment analysis of downregulated DEGs in integrated dataset. (C) This figure shows KEGG pathway enrichment analysis of upregulated DEGs in the GSE40435 dataset. (D) This figure shows KEGG pathway enrichment analysis of downregulated DEGs in the GSE40435 dataset. [file IJCB-2026-5567255-s003.zip › Supplementary Figure S2 A.pdf]

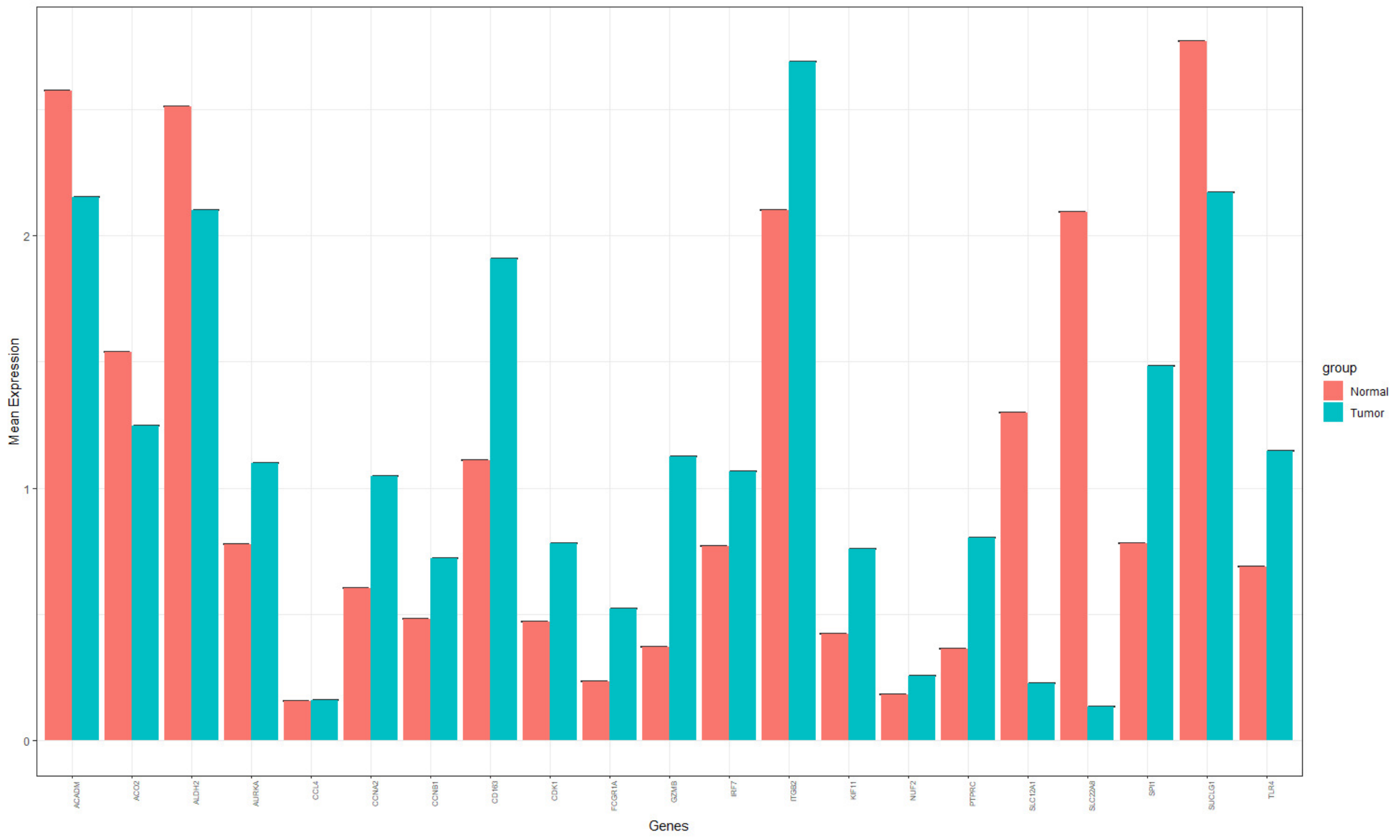

Supplement: Supplementary file 1 — Supporting Information 1 Figure S1. Volcano plots depicting differential gene expression (DEG) analysis results for each dataset, performed independently using the GEO2R online tool. Figure S2. Principal component analysis (PCA) before and after batch‐effect correction. Figure S3. Venn diagram showing the overlap of differentially expressed genes (DEGs) identified by combining p values using Fisher′s sum of logs method and by combining individual effect sizes using a random effects model. Figure S4. (A) This figure shows cellular component enrichment analysis of upregulated DEGs in integrated dataset. (B) This figure shows cellular component enrichment analysis of downregulated DEGs in integrated dataset. (C) This figure shows molecular function enrichment analysis of upregulated DEGs in integrated dataset. (D) This figure shows molecular function enrichment analysis of downregulated DEGs in integrated dataset. (E) This figure shows cellular component enrichment analysis of upregulated DEGs in the GSE40435 dataset. (F) This figure shows cellular component enrichment analysis of downregulated DEGs in the GSE40435 dataset. (G) This figure shows molecular function enrichment analysis of upregulated DEGs in the GSE40435 dataset. (H) This figure shows molecular function enrichment analysis of downregulated DEGs in the GSE40435 dataset. Figure S5 (A) This figure shows KEGG pathway enrichment analysis of upregulated DEGs in integrated dataset. (B) This figure shows KEGG pathway enrichment analysis of downregulated DEGs in integrated dataset. (C) This figure shows KEGG pathway enrichment analysis of upregulated DEGs in the GSE40435 dataset. (D) This figure shows KEGG pathway enrichment analysis of downregulated DEGs in the GSE40435 dataset. [file IJCB-2026-5567255-s003.zip › Supplementary Figure S6.pdf]

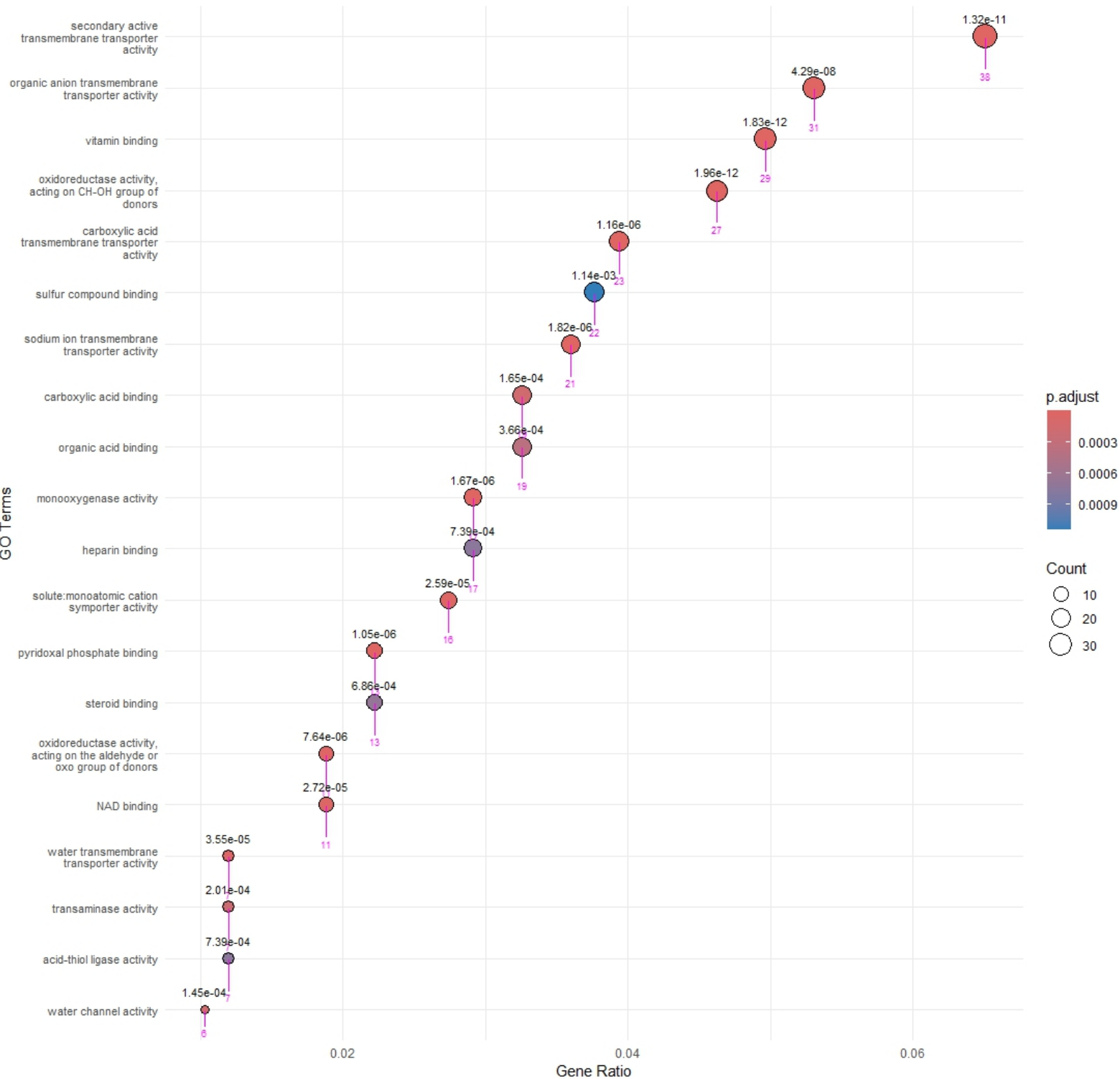

Supplement: Supplementary file 1 — Supporting Information 1 Figure S1. Volcano plots depicting differential gene expression (DEG) analysis results for each dataset, performed independently using the GEO2R online tool. Figure S2. Principal component analysis (PCA) before and after batch‐effect correction. Figure S3. Venn diagram showing the overlap of differentially expressed genes (DEGs) identified by combining p values using Fisher′s sum of logs method and by combining individual effect sizes using a random effects model. Figure S4. (A) This figure shows cellular component enrichment analysis of upregulated DEGs in integrated dataset. (B) This figure shows cellular component enrichment analysis of downregulated DEGs in integrated dataset. (C) This figure shows molecular function enrichment analysis of upregulated DEGs in integrated dataset. (D) This figure shows molecular function enrichment analysis of downregulated DEGs in integrated dataset. (E) This figure shows cellular component enrichment analysis of upregulated DEGs in the GSE40435 dataset. (F) This figure shows cellular component enrichment analysis of downregulated DEGs in the GSE40435 dataset. (G) This figure shows molecular function enrichment analysis of upregulated DEGs in the GSE40435 dataset. (H) This figure shows molecular function enrichment analysis of downregulated DEGs in the GSE40435 dataset. Figure S5 (A) This figure shows KEGG pathway enrichment analysis of upregulated DEGs in integrated dataset. (B) This figure shows KEGG pathway enrichment analysis of downregulated DEGs in integrated dataset. (C) This figure shows KEGG pathway enrichment analysis of upregulated DEGs in the GSE40435 dataset. (D) This figure shows KEGG pathway enrichment analysis of downregulated DEGs in the GSE40435 dataset. [file IJCB-2026-5567255-s003.zip › Supplementary Figure S4 H.pdf]

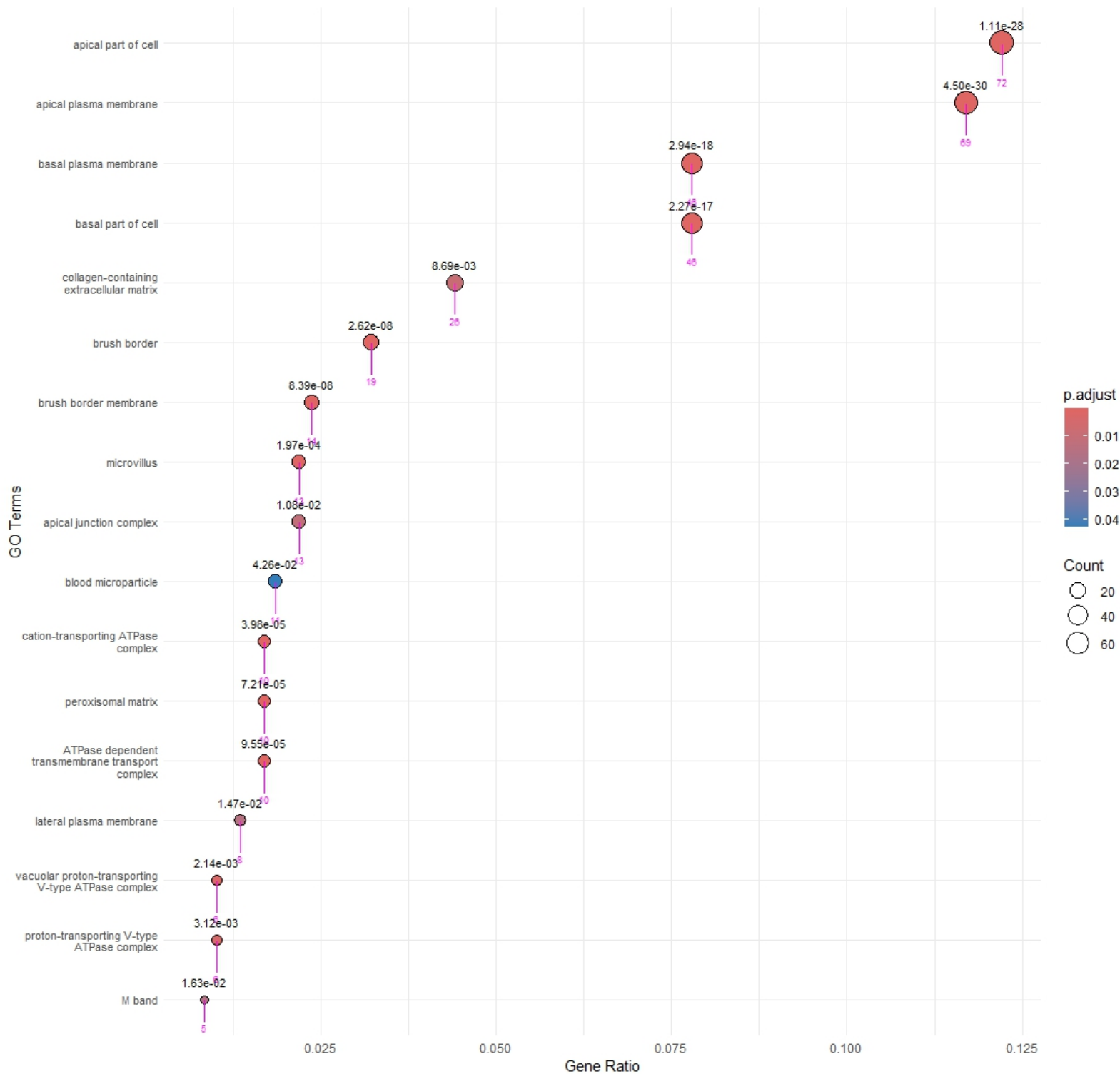

Supplement: Supplementary file 1 — Supporting Information 1 Figure S1. Volcano plots depicting differential gene expression (DEG) analysis results for each dataset, performed independently using the GEO2R online tool. Figure S2. Principal component analysis (PCA) before and after batch‐effect correction. Figure S3. Venn diagram showing the overlap of differentially expressed genes (DEGs) identified by combining p values using Fisher′s sum of logs method and by combining individual effect sizes using a random effects model. Figure S4. (A) This figure shows cellular component enrichment analysis of upregulated DEGs in integrated dataset. (B) This figure shows cellular component enrichment analysis of downregulated DEGs in integrated dataset. (C) This figure shows molecular function enrichment analysis of upregulated DEGs in integrated dataset. (D) This figure shows molecular function enrichment analysis of downregulated DEGs in integrated dataset. (E) This figure shows cellular component enrichment analysis of upregulated DEGs in the GSE40435 dataset. (F) This figure shows cellular component enrichment analysis of downregulated DEGs in the GSE40435 dataset. (G) This figure shows molecular function enrichment analysis of upregulated DEGs in the GSE40435 dataset. (H) This figure shows molecular function enrichment analysis of downregulated DEGs in the GSE40435 dataset. Figure S5 (A) This figure shows KEGG pathway enrichment analysis of upregulated DEGs in integrated dataset. (B) This figure shows KEGG pathway enrichment analysis of downregulated DEGs in integrated dataset. (C) This figure shows KEGG pathway enrichment analysis of upregulated DEGs in the GSE40435 dataset. (D) This figure shows KEGG pathway enrichment analysis of downregulated DEGs in the GSE40435 dataset. [file IJCB-2026-5567255-s003.zip › Supplementary Figure S4 F.pdf]

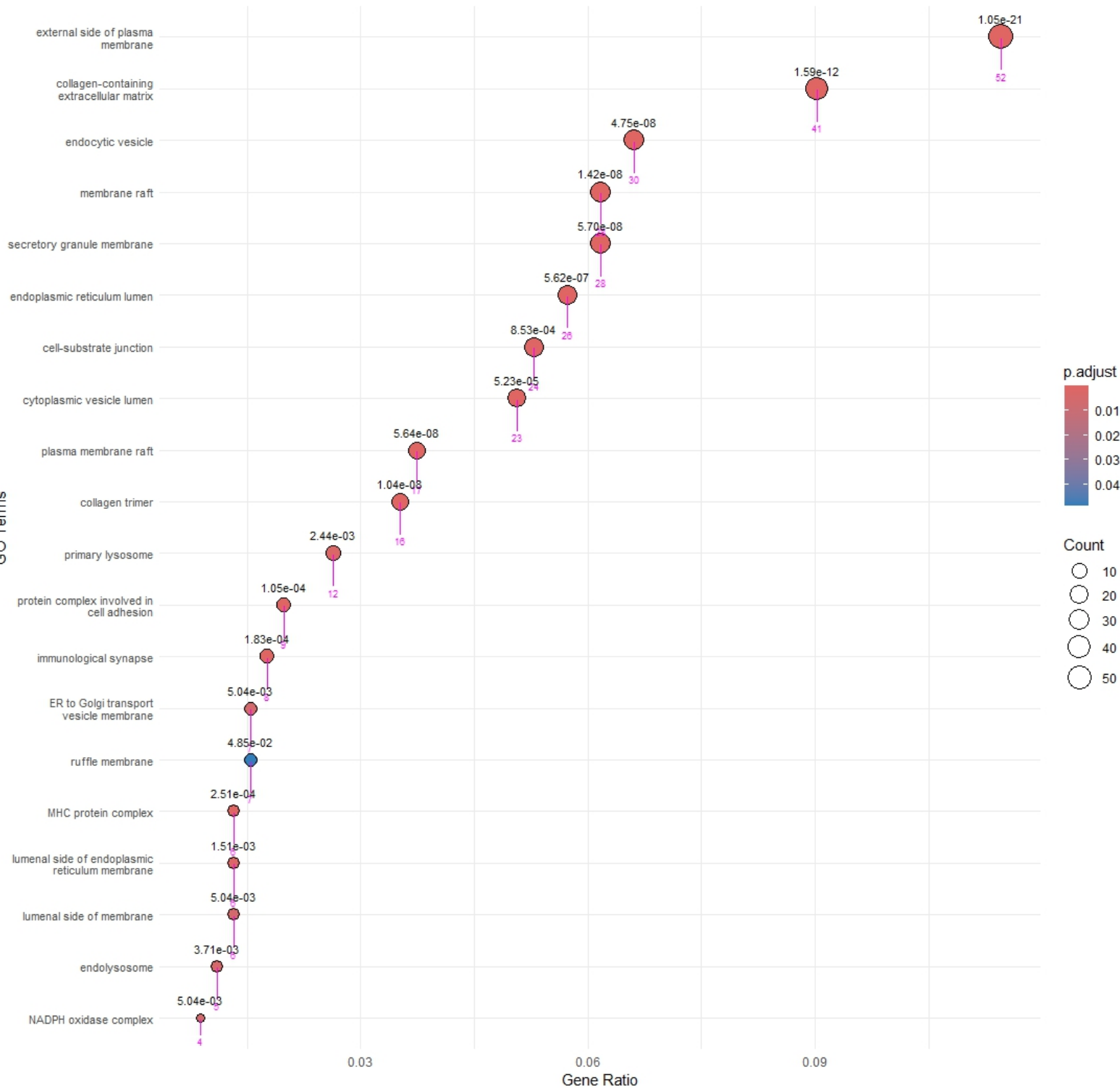

Supplement: Supplementary file 1 — Supporting Information 1 Figure S1. Volcano plots depicting differential gene expression (DEG) analysis results for each dataset, performed independently using the GEO2R online tool. Figure S2. Principal component analysis (PCA) before and after batch‐effect correction. Figure S3. Venn diagram showing the overlap of differentially expressed genes (DEGs) identified by combining p values using Fisher′s sum of logs method and by combining individual effect sizes using a random effects model. Figure S4. (A) This figure shows cellular component enrichment analysis of upregulated DEGs in integrated dataset. (B) This figure shows cellular component enrichment analysis of downregulated DEGs in integrated dataset. (C) This figure shows molecular function enrichment analysis of upregulated DEGs in integrated dataset. (D) This figure shows molecular function enrichment analysis of downregulated DEGs in integrated dataset. (E) This figure shows cellular component enrichment analysis of upregulated DEGs in the GSE40435 dataset. (F) This figure shows cellular component enrichment analysis of downregulated DEGs in the GSE40435 dataset. (G) This figure shows molecular function enrichment analysis of upregulated DEGs in the GSE40435 dataset. (H) This figure shows molecular function enrichment analysis of downregulated DEGs in the GSE40435 dataset. Figure S5 (A) This figure shows KEGG pathway enrichment analysis of upregulated DEGs in integrated dataset. (B) This figure shows KEGG pathway enrichment analysis of downregulated DEGs in integrated dataset. (C) This figure shows KEGG pathway enrichment analysis of upregulated DEGs in the GSE40435 dataset. (D) This figure shows KEGG pathway enrichment analysis of downregulated DEGs in the GSE40435 dataset. [file IJCB-2026-5567255-s003.zip › Supplementary Figure S4 E.pdf]

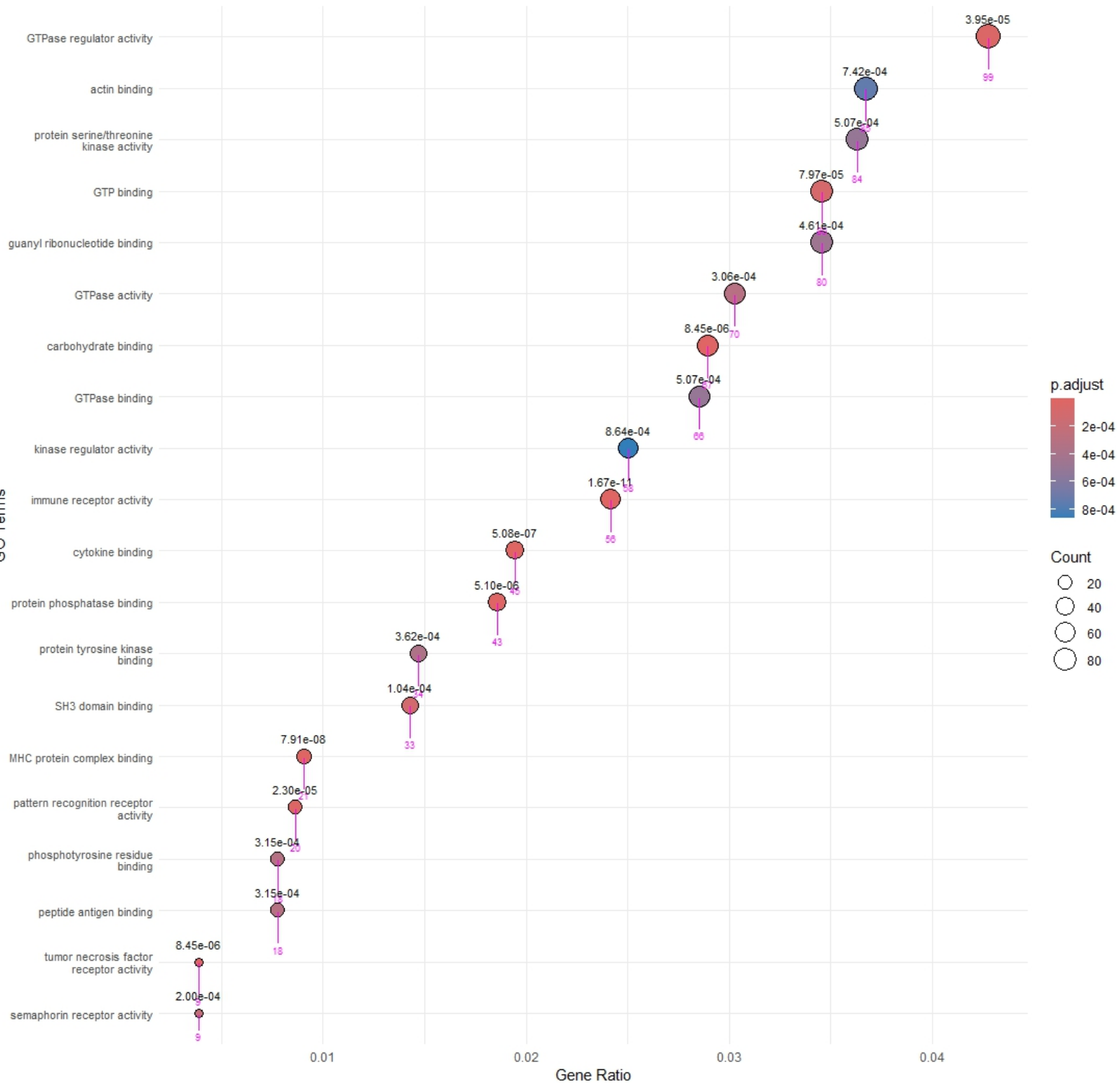

Supplement: Supplementary file 1 — Supporting Information 1 Figure S1. Volcano plots depicting differential gene expression (DEG) analysis results for each dataset, performed independently using the GEO2R online tool. Figure S2. Principal component analysis (PCA) before and after batch‐effect correction. Figure S3. Venn diagram showing the overlap of differentially expressed genes (DEGs) identified by combining p values using Fisher′s sum of logs method and by combining individual effect sizes using a random effects model. Figure S4. (A) This figure shows cellular component enrichment analysis of upregulated DEGs in integrated dataset. (B) This figure shows cellular component enrichment analysis of downregulated DEGs in integrated dataset. (C) This figure shows molecular function enrichment analysis of upregulated DEGs in integrated dataset. (D) This figure shows molecular function enrichment analysis of downregulated DEGs in integrated dataset. (E) This figure shows cellular component enrichment analysis of upregulated DEGs in the GSE40435 dataset. (F) This figure shows cellular component enrichment analysis of downregulated DEGs in the GSE40435 dataset. (G) This figure shows molecular function enrichment analysis of upregulated DEGs in the GSE40435 dataset. (H) This figure shows molecular function enrichment analysis of downregulated DEGs in the GSE40435 dataset. Figure S5 (A) This figure shows KEGG pathway enrichment analysis of upregulated DEGs in integrated dataset. (B) This figure shows KEGG pathway enrichment analysis of downregulated DEGs in integrated dataset. (C) This figure shows KEGG pathway enrichment analysis of upregulated DEGs in the GSE40435 dataset. (D) This figure shows KEGG pathway enrichment analysis of downregulated DEGs in the GSE40435 dataset. [file IJCB-2026-5567255-s003.zip › Supplementary Figure S4 C.pdf]
