# Supplementary material for: Integrative Meta‐Analysis and WGCNA Reveal Candidate Diagnostic Hub Genes in Clear Cell Carcinoma
Source: Int J Cell Biol. 2026 Feb 2;2026:5567255. doi: 10.1155/ijcb/5567255 (PMC12865128; doi:10.1155/ijcb/5567255)
Supplement: Supplementary file 3 — Supporting Information 3 Table S1. Shared DEGs identified by both statistical frameworks via Venn diagram analysis. Table S2. BP enrichment analysis of upregulated DEGs form integrated dataset using clusterProfiler. Table S3. BP enrichment analysis of downregulated DEGs form integrated dataset using clusterProfiler. Table S4. List of DEGs in the validation GSE40435 dataset. Table S5. BP enrichment analysis of upregulated DEGs in the validation GSE40435 dataset. Table S6. Biological process enrichment analysis of downregulated DEGs in the validation GSE40435 dataset. Table S7. Cellular component enrichment analysis of upregulated DEGs in integrated dataset. Table S8. Cellular component enrichment analysis of downregulated DEGs in integrated dataset. Table S9. Molecular function enrichment analysis of upregulated DEGs in integrated dataset. Table S10. Molecular function enrichment analysis of downregulated DEGs in integrated dataset. Table S11. Cellular component enrichment analysis of upregulated DEGs in the GSE40435 dataset. Table S12. Cellular component enrichment analysis of downregulated DEGs in the GSE40435 dataset. Table S13. Molecular function enrichment analysis of upregulated DEGs in the GSE40435 dataset. Table S14. Molecular function enrichment analysis of downregulated DEGs in the GSE40435 dataset. Table S15. KEGG pathway enrichment analysis of upregulated DEGs in the integrated dataset. Table S16. KEGG pathway enrichment analysis of downregulated DEGs in the integrated dataset. Table S17. KEGG pathway enrichment analysis of upregulated DEGs in the GSE40435 dataset. Table S18. KEGG pathway enrichment analysis of downregulated DEGs in the GSE40435 dataset. Table S19. Summary of transcription factors (TFs) identified among DEGs. Table S20. List of eight gene coexpression modules identified by dynamic tree cutting (β = 19, minimum size = 30 genes). Table S21. Transcription factor enrichment in the turquoise gene coexpression module. Table S22. Diagnostic biom [file IJCB-2026-5567255-s001.zip › Supplementary Table 26.docx]

| **No** | **ID** | **Degree** | **Betweenness** | **No** | **ID** | **Degree** | **Betweenness** |
| --- | --- | --- | --- | --- | --- | --- | --- |
| **1** | hsa-let-7b-5p | 12 | 706.5159 | **30** | hsa-miR-23a-3p | 8 | 307.8255 |
| **2** | hsa-miR-19a-3p | 12 | 585.856 | **31** | hsa-miR-181b-5p | 8 | 287.5989 |
| **3** | hsa-miR-19b-3p | 12 | 585.856 | **32** | hsa-miR-124-3p | 8 | 297.0365 |
| **4** | hsa-miR-34a-5p | 12 | 645.3513 | **33** | hsa-miR-146a-5p | 8 | 568.3933 |
| **5** | hsa-let-7a-5p | 11 | 554.4543 | **34** | hsa-miR-18b-5p | 8 | 293.241 |
| **6** | hsa-let-7c-5p | 11 | 528.1314 | **35** | hsa-miR-16-5p | 7 | 198.1987 |
| **7** | hsa-miR-27a-3p | 11 | 504.1675 | **36** | hsa-miR-18a-5p | 7 | 239.7189 |
| **8** | hsa-miR-196a-5p | 11 | 658.3691 | **37** | hsa-miR-30a-5p | 7 | 239.4889 |
| **9** | hsa-let-7e-5p | 10 | 442.3099 | **38** | hsa-miR-106a-5p | 7 | 250.7025 |
| **10** | hsa-miR-15a-5p | 10 | 399.5652 | **39** | hsa-miR-7-5p | 7 | 284.671 |
| **11** | hsa-miR-26a-5p | 10 | 371.4825 | **40** | hsa-miR-182-5p | 7 | 285.3482 |
| **12** | hsa-miR-26b-5p | 10 | 433.2042 | **41** | hsa-miR-205-5p | 7 | 339.6652 |
| **13** | hsa-miR-98-5p | 10 | 484.5121 | **42** | hsa-miR-210-3p | 7 | 227.7669 |
| **14** | hsa-miR-139-5p | 10 | 484.5121 | **43** | hsa-miR-221-3p | 7 | 107.3006 |
| **15** | hsa-let-7i-5p | 10 | 484.5121 | **44** | hsa-miR-15b-5p | 7 | 198.2331 |
| **16** | hsa-let-7d-5p | 9 | 388.6589 | **45** | hsa-miR-128-3p | 7 | 266.2579 |
| **17** | hsa-miR-20a-5p | 9 | 388.3214 | **46** | hsa-miR-92b-3p | 7 | 237.5013 |
| **18** | hsa-miR-92a-3p | 9 | 377.2493 | **47** | hsa-miR-33b-5p | 7 | 224.8848 |
| **19** | hsa-miR-101-3p | 9 | 221.6362 | **48** | hsa-miR-454-3p | 7 | 194.6591 |
| **20** | hsa-miR-103a-3p | 9 | 321.8413 | **50** | hsa-miR-25-3p | 6 | 144.2537 |
| **21** | hsa-let-7g-5p | 9 | 366.3155 | **51** | hsa-miR-29a-3p | 6 | 189.917 |
| **22** | hsa-miR-1-3p | 9 | 436.5293 | **52** | hsa-miR-33a-5p | 6 | 181.213 |
| **23** | hsa-miR-23b-3p | 9 | 355.343 | **53** | hsa-miR-147a | 6 | 198.9585 |
| **24** | hsa-miR-106b-5p | 9 | 388.3214 | **54** | hsa-miR-181a-5p | 6 | 193.1283 |
| **25** | hsa-miR-424-5p | 9 | 433.369 | **55** | hsa-miR-30e-3p | 6 | 176.058 |
| **26** | hsa-miR-423-5p | 9 | 448.5828 | **56** | hsa-miR-20b-5p | 6 | 166.2714 |
| **27** | hsa-let-7f-5p | 8 | 330.5465 | **57** | hsa-miR-449a | 6 | 181.1765 |
| **28** | hsa-miR-17-5p | 8 | 314.1461 | **58** | hsa-miR-374b-5p | 6 | 193.2665 |
| **29** | hsa-miR-21-5p | 8 | 429.9959 | **60** |  |  |  |

**Supplementary Table 26.** List of miRNAs included in the miRNA–mRNA regulatory network, limited to those with a degree of 6 or higher.
